# Synthesis and biological evaluation of novel 1,2,3-triazole hybrids of cabotegravir: identification of potent antitumor activity against lung cancer

**DOI:** 10.3389/fphar.2023.1265245

**Published:** 2023-09-20

**Authors:** Yajie Guo, Dan Sang, Bin Guo, Dan Wang, Xinyue Xu, Huili Wang, Cuilan Hou, Longfei Mao, Fang Li, Sanqiang Li

**Affiliations:** ^1^ Department of Emergency, The Eighth Affiliated Hospital, Sun Yat-Sen University, Shenzhen, China; ^2^ Department of Endocrinology, The Eighth Affiliated Hospital, Sun Yat-Sen University, Shenzhen, China; ^3^ Ultrasonic Department, The First Affiliated Hospital of USTC, Division of Life Sciences and Medicine, University of Science and Technology of China, Hefei, Anhui, China; ^4^ School of Public Health, Southern Medical University, Guangzhou, China; ^5^ Shenzhen Center for Disease Control and Prevention, Shenzhen, China; ^6^ School of Public Health, University of South China, Hengyang, Hunan, China; ^7^ University of North Carolina Hospitals, Chapel Hill, NC, United States; ^8^ Department of Cardiology, Shanghai Children’s Hospital, Shanghai Jiaotong University, Shanghai, China; ^9^ College of Basic Medicine and Forensic Medicine, Henan University of Science and Technology, Luoyang, China; ^10^ State Key Laboratory of Medicinal Chemical Biology, College of Pharmacy and Tianjin Key Laboratory of Molecular Drug Research, Nankai University, Tianjin, China; ^11^ Hainan Women and Children’s Medical Center, Affliated Children’s Hospital of Hainan Medical University, Haikou, Hainan Province, China

**Keywords:** 1,2,3-triazoles, cabotegravir, anticancer, lung cancer, apoptosis, ROS

## Abstract

In pursuit of discovering novel anticancer agents, we designed and synthesized a series of novel 1,2,3-triazole hybrids based on cabotegravir analogues. These compounds were subjected to initial biological evaluations to assess their anticancer activities against non-small-cell lung cancer (NSCLC). Our findings indicated that some of these compounds exhibited promising antitumor abilities against H460 cells, while demonstrated less efficacy against H1299 cells. Notably, compound **5i** emerged as the most potent, displaying an IC_50_ value of 6.06 μM. Furthermore, our investigations into cell apoptosis and reactive oxygen species (ROS) production revealed that compound **5i** significantly induced apoptosis and triggered ROS generation. Additionally, Western blot analysis revealed the pronounced elevation of LC3 expression in H460 cells and γ-H2AX expression in H1299 cells subsequent to treatment with compound 5i. These molecular responses potentially contribute to the observed cell death phenomenon. These findings highlight the potential of compound **5i** as a promising candidate for further development as an anticancer agent especially lung cancer.

## 1 Introduction

Lung cancer is the leading cause of cancer-related deaths worldwide and advanced non-small-cell lung cancer (NSCLC) is the most common type. Surgical intervention is the preferred treatment for patients with stage I-II non-small-cell lung cancer (NSCLC), while chemotherapy combined with radiotherapy is recommended for stage III NSCLC patients (Auperin et al., 2010; Curran et al., 2011; Vansteenkiste et al., 2014). Approximately 69% of patients with advanced NSCLC harbor specific molecular aberrations, presenting a spectrum of actionable targets. This encompasses kinds of key regulators including EGFR, ALK, ROS1, BRAF, MET, RET, and HER2. To tackle these diverse targets, a corresponding arsenal of marketed drugs has emerged, such as erlotinib, osimertinib, dacomitinib, lorlatinib, TPX-0005, capmatinib, and so on (Tsao et al., 2016). These make it possible for a number of patients to have an improved long-term survival. For years, people devoted their efforts in finding drugs either offer better alternatives to current standard drugs or are completely new with novel actions. Despite the development of anticancer drugs, there is an ongoing need to discover and design safer and more effective agents with reduced side effects and drug resistance.

**FIGURE 1:**
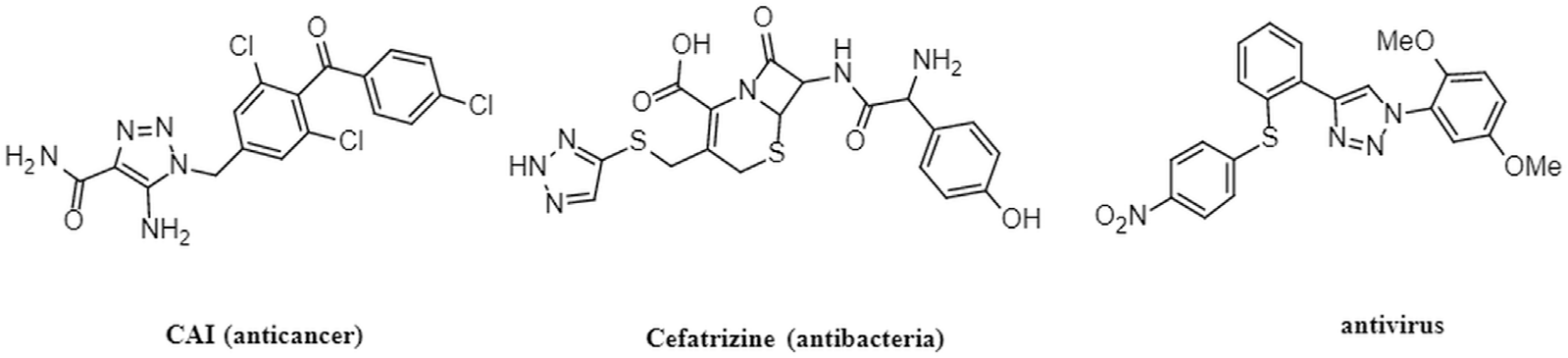
Structures of 1,2,3-triazole drugs with biological effects.

Heterocycles play an important role in the process of drug discovery and design (Markwardt, 1978). Among them, 1,2,3-triazoles are nitrogen containing heterocycles that have three nitrogen atoms in the ring, making them less susceptible to metabolic degradation compared to other heterocycles (Dalvie et al., 2002). The incorporation of 1,2,3-triazole units into drug molecules using click chemistry approaches has gained significant popularity due to their ability to form stable compounds with hydrogen bonding interactions, enhancing drug solubility and efficacy (Kolb and Sharpless, 2003). Consequently, drugs containing 1,2,3-trazole structures have been developed and reported to possess diverse biological properties, including anticancer (Yang et al., 2020; Hasanpour et al., 2021), antimicrobial (Lal et al., 2018; Addo et al., 2022), antiviral (Kutkat et al., 2022), and anti-HIV activities (Feng et al., 2021). Figure 1 illustrates examples of drugs containing 1,2,3-triazole structures with different biological activities. Recently, researchers have investigated new class of 1,2,3-triazole hybrids that have pharmacological activities. For example, isatin-semicarbazone tethered 1,2,3-triazole compounds, which were synthesized via Cu-mediated azide alkyne cycloaddition reaction, were reported to be efficacy towards *E. coli* with MIC of 0.0063 μmol/mL (Kumar et al., 2023). 1,2,3-triazole analogue compounds with isoxazole replaced with triazole core could relieve mitochondrial dysfunction which was useful in treating cardiovascular diseases (Algieri et al., 2023). 1,2,3-triazole hybrids combined with various natural compounds, chalcone hybrids, carbohydrate or azole hybrids were found to have anticancer activity that were effective to different cancers such as lung cancer, prostate cancer, breast cancer, and colon cancer (Alam, 2022).

**FIGURE 2:**
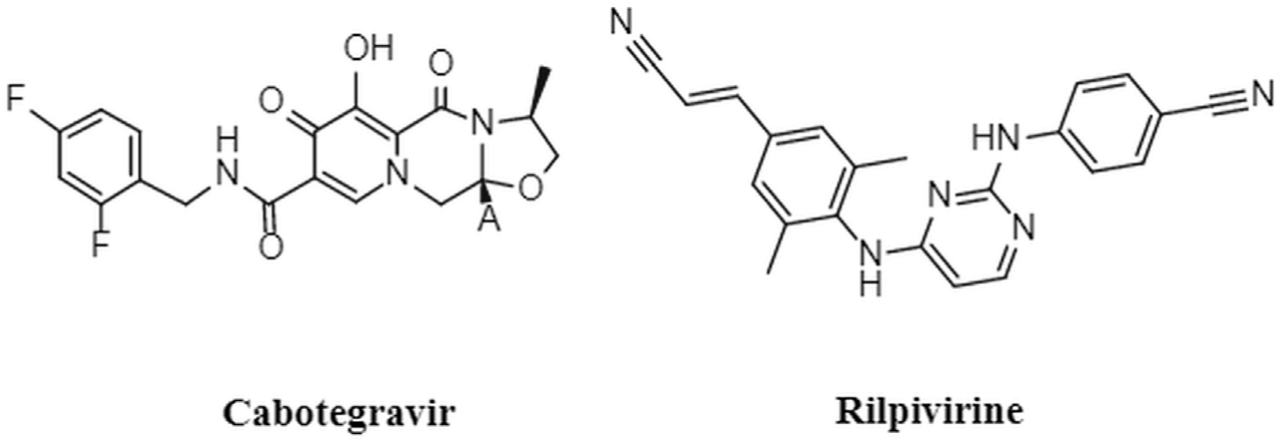
Structures of cabotegravir and rilpivirine.

Notably, Cabenuva (cabotegravir/rilpivirine) (Figure 2) is the latest FDA-approved injectable combination therapy for HIV treatment, comprising a long-acting formulation of cabotegravir (CAB) and rilpivirine (RPV) (Durham and Chahine, 2021). CAB, a second generation integrase strand transfer inhibitor, and RPV, a non-nucleoside reverse transcriptase inhibitor, were jointly developed by ViiB Healthcare and Janssen Pharmaceutica (Markham, 2020). This medication is indicated for adults aged 18 years and older who have achieved viral suppression through an oral treatment regimen for at least 6 months. Additionally, it is intended for those who have no prior instances of treatment failure and exhibit no resistance to CAB or RPV (Rial-Crestelo et al., 2020). Cabotegravir/rilpivirine exhibits potent activity against a wide range of HIV subtypes and inhibits HIV integrase-catalyzed chain transfer with an IC_50_ of 3 nM. Clinical trials have demonstrated its efficacy and high patient satisfaction among individuals living with HIV (Andrews and Heneine, 2015; Reese et al., 2016).

In recent years, efforts have been continuously directed towards the development of safer and less side-effect anticancer drugs. Repurposing mature drugs that are already on the market and evaluating their potential anticancer effects is an important approach in anticancer drugs development. This ensures both drug safety and clinical applicability. Integrating 1,2,3-triazole hybrids with known drugs has become a popular strategy for anticancer drug discovery. Preliminary researches have revealed promising anticancer effects by combining the 1,2,3-triazole hybrids with natural compounds such as betulin, myrrhanone B, coumarin, hydnocarpic acid, curcumin and others (Alam, 2022). While cabotegravir is an effective drug for treating HIV/AIDS, there has been no study investigating the combination of 1,2,3-triazole hybrids and cabotegravir for anticancer purposes. Building upon our previous research focusing on the synthesis of 1,2,3-triazole analogues with known drugs, which exhibited promising anticancer activity against human cancer cell lines, we sought to explore the antitumor activity of cabotegravir and 1,2,3-traizoles combination. In this study, we designed and synthesized a novel series of molecular hybrids that merge these two molecular fragments. Subsequently, we conducted a comprehensive evaluation of their biological activity especially the antitumor activity against lung cancer cell lines, with the aim of discovering more efficient anticancer agents for treating lung cancer.

## 2 Materials and methods

### 2.1 Chemistry

Elemental analyses were conducted using an Elementar Analysensystem Vario Macro cube in CHNSO mode. NMR spectra analyses were recorded on a Bruker400 spectrometer. ^1^H NMR and ^13^C NMR spectra were performed in DMSO-d_6_ solution. Chemical shifts d) were reported in parts per million with tetramethylsilane as internal reference, and coupling constants were expressed in hertz using the following abbreviations: s (singlet), d (doublet), t (triplet), m (multiplet).

#### 2.1.1 Synthesis of compound 2 (1-(formylmethyl)-5-methoxy-6-(methoxycarbonyl)-4-oxopyridine-3-carboxylic acid)

This step is a classic reaction of the demethylation of dimethyl aldehyde under acidic conditions. 1-(2,2-dimethoxylethyl)-1,4-dihydro-3-methoxy-4-oxo-2,5-pyridinedicarboxylic acid-2-methyl ester (compound **1**) (310 g, 1.0 mol) was combined with anhydrous formic acid (1,000 mL). The reaction mixture was heated to 65°C and allowed to react for 3 h under an argon atmosphere. The progress of the reaction was monitored using TLC (V(Petroleum ether):V(ethyl acetate) = 3:1, RF = 0.3–0.6). Afterward, the reaction mixture was concentrated under vacuum at 45°C to eliminate the formic acid, resulting in the formation of a crude oil compound. To remove any residual formic acid, acetonitrile (300 mL) was added, and the solvent was subsequently evaporated under vacuum. This process led to the isolation of compound **2** in the form of an oil.

#### 2.1.2 Synthesis of compound 3 (3S,11aR)-6-methoxy-3-methyl-5,7-dioxo-3,5,11,11a-tetrahydro-2H-[1,3]oxazolo[3,2-d]pyrido[1,2-a]pyrazine-8-carboxylic acid)

The mechanism of this step involves the condensation of compound **2** with S-amino-propanol, leading to the formation of an intermediate compound, denoted as compound **a**. Compound **a** undergoes intramolecular cyclization to form intermediate compound **b**. The imine group within compound **b** then undergoes condensation with the ester group to yield compound **3**. The mechanism of this step is documented in the previous article (Johns et al., 2013).

Compound **2** was dissolved in acetonitrile (1,000 mL), and then S-2-aminopropanol (125 g, 1.4 mol) was added to the solution. The reaction mixture was heated to 80°C–82°C and stirred for 2 h. The progress of the reaction was monitored using TLC (V(Petroleum ether):V(ethyl acetate) = 3:1, RF = 0.3–0.6). The mixture was then concentrated under 45°C, and dichloromethane (2000 mL) was added. After complete mixing, water (1,000 mL) was added, followed by the addition of 2 N hydrochloric acid to adjust the PH to the range of 1–2. The lower organic phase was separated and the upper aqueous phase was extracted three times with methylene chloride (500 mL). Combined with the organic phase, washed these using saturated salt water (400 mL) for three times. The resulting crude product, compound **3** (165 g), was further purified using methyl alcohol. ^1^H NMR (400 MHz, DMSO) δ 15.46 (d, J = 8.1 Hz, 1H), 8.75 (d, J = 3.2 Hz, 1H), 5.43 (d, J = 82.2 Hz, 1H), 4.87 (d, J = 80.4 Hz, 1H), 4.41 (d, J = 47.8 Hz, 1H), 4.14 (d, J = 53.8 Hz, 2H), 3.89 (d, J = 4.2 Hz, 3H), 3.72 (dd, J = 10.8, 5.9 Hz, 1H), 3.14 (d, J = 85.1 Hz, 1H), 1.33 (d, J = 29.1 Hz, 3H).

#### 2.1.3 Synthesis of compound 4 (dolutegravir-terminal alkyne derivative)

This step is a classic reaction involving the condensation of a carboxyl group and an amino group. Compound **3** (30 g), HATU (38 g), DIPEA (25 g), aminophenylacetylene (23 g), and N,N-dimethylformamide (1500 mL) was mixed at room temperature, and reacted for 12 h. Water (500 mL) was added, and the mixture was stirred, filtered, and dried overnight to obtain compound **4** (34 g), yield 86.5%. ^1^H NMR (400 MHz, DMSO) δ 12.53 (s, 1H), 8.69 (s, 1H), 7.95 (s, 1H), 7.58 (s, 1H), 7.38 (s, 1H), 7.22 (s, 1H), 5.42 (d, J = 78.6 Hz, 1H), 4.87 (d, J = 76.4 Hz, 1H), 4.40 (d, J = 25.2 Hz, 1H), 4.14 (d, J = 68.8 Hz, 2H), 3.80 (d, J = 72.6 Hz, 3H), 3.13 (d, J = 67.9 Hz, 1H), 2.81 (d, J = 63.8 Hz, 1H), 1.29 (d, J = 48.7 Hz, 3H).

#### 2.1.4 Synthesis of compounds 5a-5s, 6a-6i

This step is the classic of a click compound reaction, which has been previously reported in articles (Asemanipoor et al., 2020). Azide compounds (0.5 g), compound **4** (0.5 g), TBA (10 mL), water (10 mL), tetrahydrofuran (10 mL), chalcanthite (0.25 g), and sodium ascorbate (0.5 g) were mixed and reacted at 70°C. After reacted completely, dichloromethane (20 mL) was added, and the reaction was then filtered. The aqueous phase was extracted twice with dichloromethane, and the organic phase was combined, dried using anhydrous magnesium sulfate, followed by evaporated. The obtained residues was subsequently recrystallized using methanol, resulting in the final isolation of the target compounds **5a-5s**, **6a-6i**.

### 2.2 Biology

For biological experiments, Dulbecco’s modified Eagle medium (DMEM), RPMI 1640 Medium, Fetal bovine serum (FBS) and penicillin/streptomycin were bought from Gibco (Grand Island, NY, United States). Dimethylsulfoxide (DMSO) was got from Sigma-Aldrich (St. Louis, Missouri, United States). Enhanced Cell Counting Kit-8, Reactive Oxygen Species (ROS) Assay Kit, Calcein/PI Live/Dead Viability Assay Kit and Giemsa dye were obtained from Beyotime Biotechnology (Shanghai, China). Annexin V-FITC/Propidium iodide (PI) staining kit was bought form BD Biosciences (Franklin Lake, New Jersey, United States).

#### 2.2.1 Cell culture

Human lung cancer cell line H460 and H1299, human liver normal cell line LO2 were bought from ATCC. DMEM or RPMI 1640 was used as the cell culture medium with 10% FBS and 1% penicillin/streptomycin. Cells were maintained in an incubator with 37°C humidified atmosphere and 5% CO_2_.

#### 2.2.2 Cell viability and IC_50_ measurement

Cell viability and IC_50_ values were determined using the CCK8 assay kit. Cells were seeded at a density of 5 × 10^3^ cells per well in a 96-well plate. After overnight adhesion, 25 μM of compounds or vehicle control DMSO which all dissolved in the culture medium were added to cells and cultured for 48 h or 72 h. CCK8 reagent, dissolved in the medium, was then added to cells and incubated for 1 h. The absorbance at 450 nm was measured using a microplate spectrophotometer (Thermo). Cell viability was calculated, with the untreated group taken as 100%. For IC_50_ values determination, different concentrations (0, 0.4 μM, 2 μM, 10 μM, 20 μM, 50 μM) of compounds were added to cells for 72 h. Subsequent CCK8 measurement allowed the calculation of inhibition percentage based on cell viability. The IC_50_ values were computed by the prism statistical software. The CCK-8 assay was conducted three times and the repetitions in each time were at least three. All the values were expressed as Mean ± SD, *p* < 0.05 was considered statistically significant.

#### 2.2.3 Plate clone formation assay

Cells with the density of 500 cells per well were seeded in a 6-well plate. Different concentrations (0, 2 μM, 4 μM, 8 μM, 16 μM and 32 μM) of compounds were added to cells and maintained for a 72- hour duration. And cells were cultured until the monoclonal appeared. Afterward, cells were fixed with 4% paraformaldehyde and stained by Giemsa dye. The plate clone numbers were photographed and counted using a microscope. Values were expressed as Mean ± SEM, *p* < 0.05 was considered statistically significant. Data were performed using Graph Prim 7.0.

#### 2.2.4 Live and dead cells staining

Cells with a density of 5 × 10^3^ cells per well were seeded in a 96-well plate. After overnight incubation, cells were treated with different concentrations (0, 5 μM, 10 μM, 20 μM) of compounds for 24 h. The LIVE/DEAD Assay Kit was used to dye cells, and cells were observed and photographed under a fluorescent microscope. Live and Dead cell numbers were counted and the ratio of dead to living cells were calculated. Values were expressed as Mean ± SEM, *p* < 0.05 was considered statistically significant. Data were performed using Graph Prim 7.0.

#### 2.2.5 Apoptosis assay

Cells with a density of 3 × 10^5^ cells per well were seeded in 6-well plates. After overnight adhesion, cells were treated with different concentrations of compounds for 72 h. Cells were harvested and stained with Annexin V-FITC and propidium iodide. Apoptotic ratio was quantified using the flow cytometry (BD Biosciences) and analyzed using the FlowJo software v10. Values were expressed as Mean ± SD, *p* < 0.05 was considered statistically significant. Data were performed using Graph Prim 7.0.

#### 2.2.6 Cellular ROS measurement

Cells with a density of 5 × 10^3^ cells/well were cultured in a 96-well plate. The cells were treated with different concentrations (0, 5, 10, 20 μM) of compounds for 24 h. Then the medium was replaced with 10 μM DCFH-DA dissolved in culture medium and incubated for 30min at 37°C in dark. The fluorescent cells were observed and photographed using a fluorescent microscope.

#### 2.2.7 Western blot

Western blot was used to analyze protein expressions. Cells were cultured in 12-well plates and treated with different concentrations (0, 2, 4, 8 μM) of compounds for 72 h. Cell proteins were extracted using RIPA buffer with protease/phosphatase inhibitor cocktail (CST). 10%–15% sodium dodecyl sulfate polyacrylamide gel electrophoresis was used to separate proteins and nitrocellulose membranes (Millipore) was used to collected proteins. Antibodies used in western bolt were LC3 (3868s, CST), caspase 3 (9662s, CST), cyclin D (2922s, CST), cyclin E (20808s, CST), β-Catenin (9562s, CST), γH2AX (9718s, CST), PARP (46D11, CST), and β-actin (4967s, CST). Values were expressed as Mean ± SEM, *p* < 0.05 was considered statistically significant. Data were performed using Graph Prim 7.0.

## 3 Results and disscussion

### 3.1 Chemistry

**SCHEME 1:**
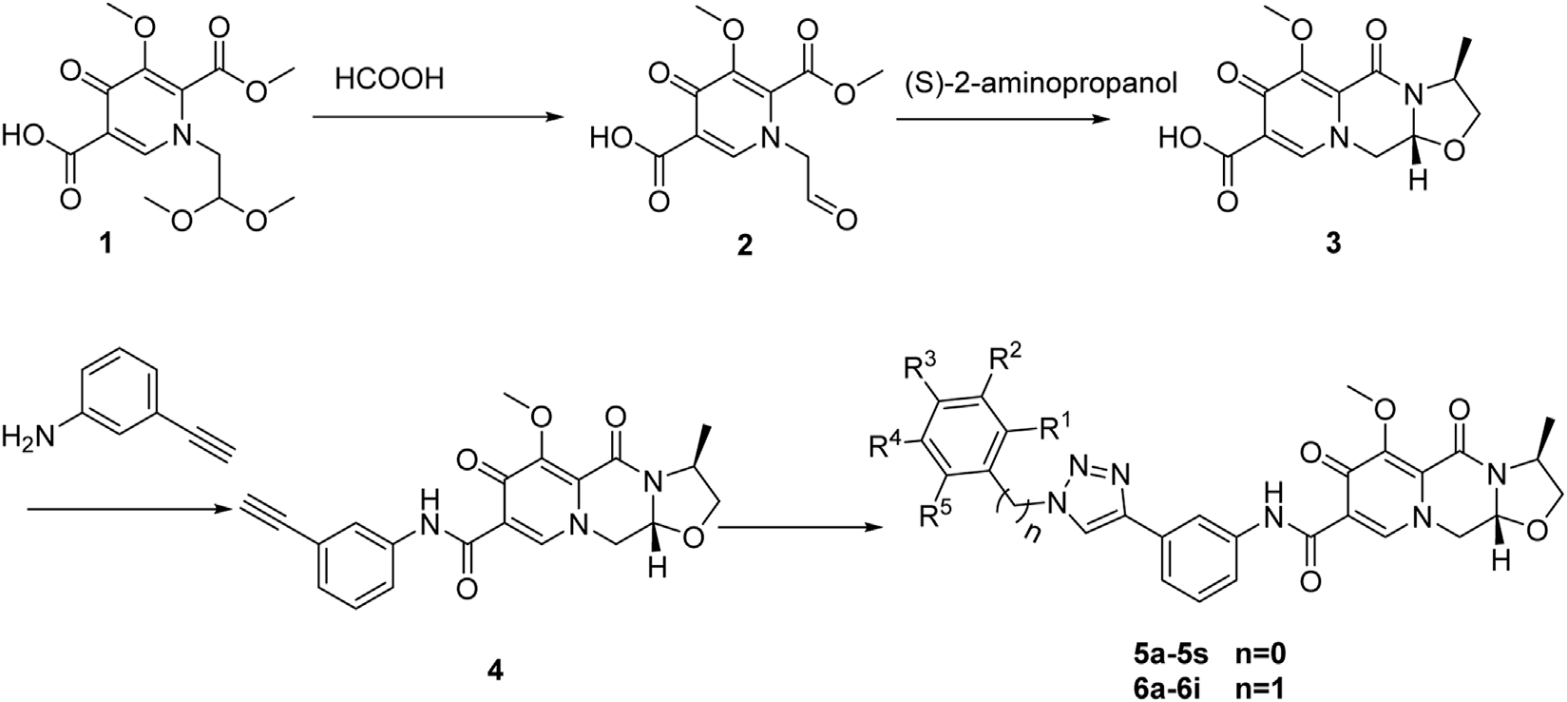
Synthesis of 1,2,3-triazole hybrids of cabotegravir derivatives.

The synthesis processes, as illustrated in Scheme 1, commenced with the hydrolysis of 1-(2,2-dimethoxyethyl) -5-methoxy-6-(methoxycarbonyl)-4-oxopyridine -3-carboxylic acid (compound **1**) using formic acid, resulting in the formation of 1-(formylmethyl)-5-methoxy-6-(methoxycarbonyl)-4-oxopyridine-3-carboxylic acid (compound **2**). Subsequently, the formic acid was removed by direct vacuum concentration without any additional treatments. Compound **2** was then subjected to a reflux reaction with acetonitrile and (S)-2-aminopropanol, leading to the formation of (3S,11aR)-6-methoxy-3-methyl-5,7-dioxo-3,5,11,11a-tetrahydro-2H-[1,3]oxazolo[3,2-d]pyrido[1,2-a]pyrazine-8-carboxylic acid (compound **3**). Using HATU and DIPEA as coupling agents, compound **3** reacted with 3-aminophenylacetylene to yield terminal alkynes compound **4**. Subsequently, compound **4** was subjected to reaction with azides containing various substituents, resulting in the synthesis of 28 novel target compounds, namely, **5a-5s** and **6a-6i**. The structures of these newly synthesized compounds are presented in Table 1. All compounds were purified using column chromatography and extensively characterized using spectral analysis techniques, including ^1^H NMR, ^13^C NMR, elemental analyses, and MS (Chen et al., 2022b). Detail information regarding the ^1^H NMR and ^13^C NMR spectrums can be found in the Supplementary Files.

**TABLE 1:** Structures of compounds **5a-5s** and **6a-6i**.

Compd no.	n	R^1^	R^2^	R^3^	R^4^	R^5^	Compd no.	n	R^1^	R^2^	R^3^	R^4^	R^5^
5a	0	CF_3_	H	H	H	H	5o	0	I	H	H	H	H
5b	0	F	H	NO_2_	H	H	5p	0	H	H	H	H	H
5c	0	CH_3_	H	H	H	H	5q	0	H	Cl	H	H	H
5d	0	CH_2_CH_3_	H	H	H	H	5r	0	H	Br	H	H	H
5e	0	H	CF_3_	H	CF_3_	H	5s	0	Cl	H	H	H	H
5f	0	H	F	H	H	H	6a	1	H	H	H	H	H
5g	0	OCH_3_	H	H	H	H	6b	1	Br	H	H	H	H
5h	0	H	CF_3_	H	H	H	6c	1	CH_3_	H	H	H	H
5i	0	OCF_3_	H	H	H	H	6d	1	H	H	CF_3_	H	H
5j	0	H	H	CF_3_	H	H	6e	1	CF_3_	H	H	H	H
5k	0	CF_3_	H	H	CF_3_	H	6f	1	H	H	Cl	H	H
5l	0	CH_3_	H	CH_3_	H	CH_3_	6g	1	H	OCH_3_	H	H	H
5m	0	Br	H	H	H	H	6h	1	Cl	H	H	H	H
5n	0	H	H	F	H	H	6i	1	H	F	H	H	H

### 3.2 Biology

#### 3.2.1 Cell viability and cytotoxicity assay

In order to assess the potential cytotoxicity of the synthesized compounds, we selected two types of human non-small-cell lung cancer cell line, H460 and H1299. Cell viability and cytotoxicity were evaluated using the CCK8 assay (Xu et al., 2018). The cells were treated with 25 μM of compounds **5a-5s** and **6a-6i** for 48h and 72 h. The results, presented in Table 2, indicated that after 48 h of treatment, the compounds showed limited anti-proliferative effects against H460 and H1299 cells. Among them, compounds **5c**, **5i**, and **6h** exhibited cell viability rates below 50% in H460 cells. However, against H1299 cells, all compounds exhibited cell viability rates exceeding 90%, indicating minor effects on this cell line. Upon extending the treatment period to 72 h, some compounds exhibited heightened cytotoxicity. Specifically, in H460 cells, compounds **5c**, **5d**, **5i**, **5s**, **6b**, and **6h** showed cell viability rates below 50%. In contrast, no compounds managed to reduce H1299 cell viability to less than 50%, yet compounds **5i**, **5m**, **6h** and **6i** achieved cell viability rates below 70%. These findings suggested that the synthesized compounds exhibited more pronounced cytotoxic effects against H460 cells compared to H1299 cells. Notably, compound **5i** displayed the most significant cytotoxicity against both cell lines. It is worth noting that minimal effects on the cell viability of the normal cell line LO2 were observed when exposed to these compounds. Further, we determined the half-maximal inhibitory concentration (IC_50_) of selected compounds against H460 cells, the results of which were summarized in Table 3. Remarkably, compound **5i** exhibited the most potent cytotoxicity with an IC_50_ value of 6.06 μM, followed by compounds **5c** and **5s** with IC_50_ values of 9.65 μM, and 13.38 μM, respectively.

**TABLE 2:** Cell viability (100%) of compounds **5a-5s** and **6a-6i** against lung cancer cell lines.

No.	H460		H1299		LO2
	48 h	72 h	48 h	72 h	72 h
5a	87.78	65.75	121.35	102.55	105.28
5b	88.35	93.31	104.47	99.70	105.92
5c	27.73	18.06	94.81	83.51	73.79
5d	92.17	19.26	117.48	99.81	127.81
5e	101.34	101.97	103.75	99.34	129.12
5f	93.31	100.16	114.19	100.41	130.35
5g	82.94	99.43	117.81	103.45	128.56
5h	97.75	75.09	114.98	93.73	101.34
5i	7.79	5.94	90.47	71.12	99.80
5j	100.66	111.72	96.98	92.49	108.63
5k	94.55	52.15	113.47	91.70	124.51
5l	84.74	98.25	118.92	91.44	125.46
5m	96.50	94.59	112.42	73.19	111.3
5n	99.22	105.04	115.05	91.74	127.86
5o	101.09	104.29	120.83	86.67	100.11
5p	88.36	104.18	114.26	87.16	109.72
5q	99.40	104.14	107.23	88.40	125.35
5r	99.03	107.13	117.54	89.82	127.81
5s	71.73	39.73	127.14	97.37	122.75
6a	99.81	118.33	118.73	101.39	127.44
6b	51.88	38.88	104.60	101.92	126.52
6c	105.51	101.80	110.58	97.63	103.41
6d	113.95	105.14	113.99	93.95	104.58
6e	77.85	60.74	127.00	92.15	127.55
6f	91.99	94.49	104.20	96.47	130.96
6g	93.36	95.01	91.06	94.37	130.54
6h	42.57	31.63	126.87	73.75	88.86
6i	97.90	95.51	99.47	64.96	127.14

**TABLE 3:** IC_50_ values (μM) of compounds **5** and **6** against H460.

No.	IC_50_	No.	IC_50_
5a	31.79 ± 0.47	5q	>100
5c	9.65 ± 0.04	5s	13.38 ± 4.54
5d	12.74 ± 4.85	6a	>50
5e	>100	6b	30.53 ± 2.18
5h	>100	6d	24.62 ± 0.82
5i	6.06 ± 1.80	6e	22.88 ± 0.13
5k	36.02 ± 2.73	6h	15.86 ± 0.09
5m	>50		

#### 3.2.2 Live/dead cell staining and plate clone formation measurement

**FIGURE 3:**
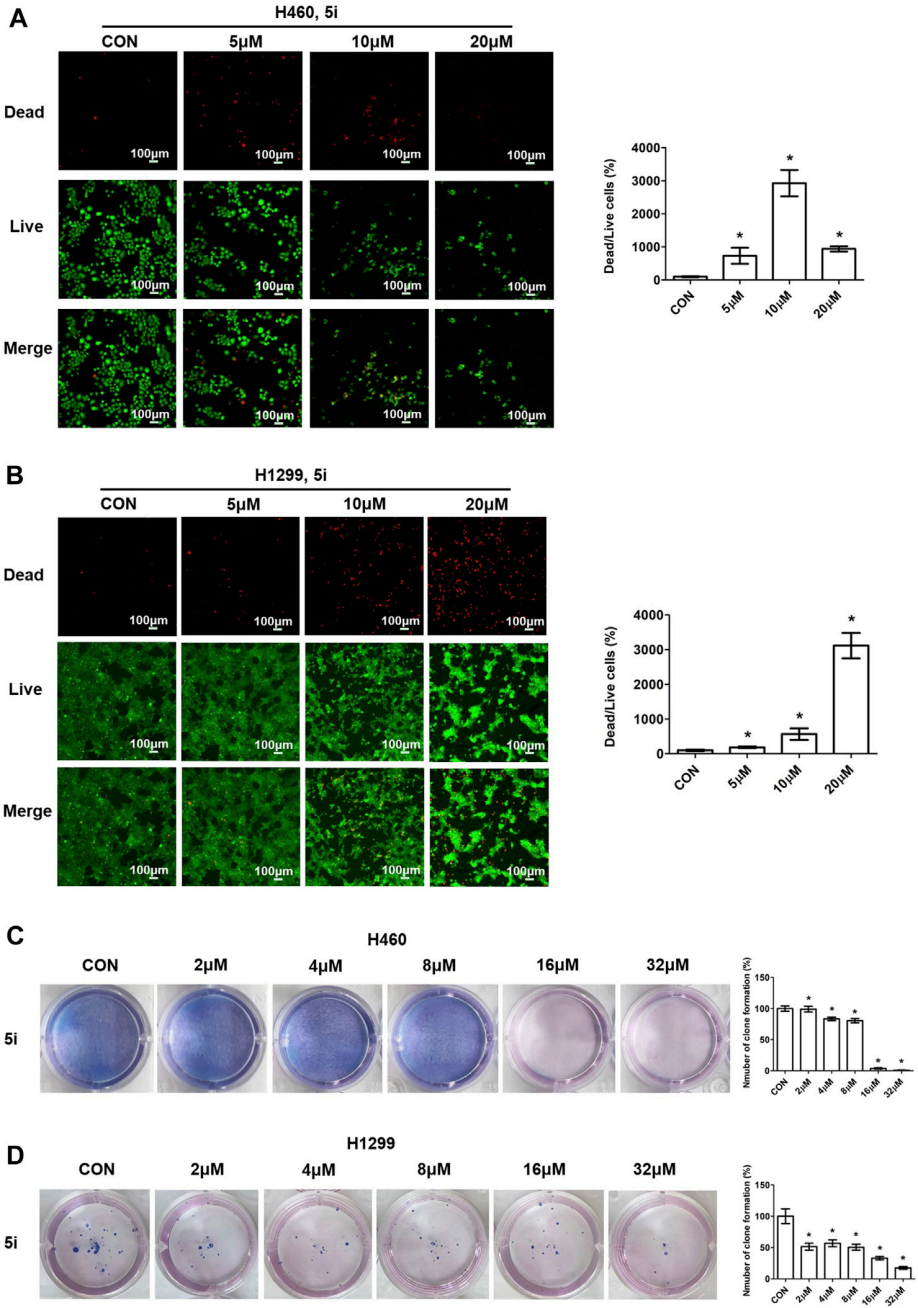
Live/dead cell staining and plate clone formation measurement of compound **5i** in H460 and H1299 cells with different concentrations. **(A, B)** Fluorescence images; **(C, D)** Plate clone formation. Left, images; right, statistical chart. Data are presented as Mean ± SEM. *p < 0.05.

To assess the anti-proliferative effects of compound **5i**, live/dead cell staining was performed using Calcein Acetoxymethyl Ester (Calcein-AM) and Propidium Iodide (PI) dyes (Xie et al., 2023). Calcein-AM highlights live cells through green fluorescence emission, while PI identifies dead cells with damaged membrane integrity, producing red fluorescence. H460 and H1299 cells were treated with different concentrations (0, 5, 10 and 20 μM) of compound **5i** for 24 h. After 24 h treatment, cells were stained with Calcein-AM and PI, diluted in cultured medium, and incubated for an hour in dark. Then the fluorescence was detected and photographed using a fluorescent microscope. The results demonstrated that compound **5i** significantly decreased the number of live cells and increased the number of dead cells in H460 cells (Figure 3A). In the 10 and 20 μM concentration groups, the number of live cells was dramatically reduced compared to the untreated group, and it showed less dead cells because of the largely decreased live cells in 20 μM concentration group (Figure 3A). Similarly, the same trends were observed in H1299 cells, as treatment with compound **5i** led to a decrease in live cell numbers alongside a substantial rise in dead cells (Figure 3B). In addition, we also performed plate clone formation assay (Xu et al., 2018) to further investigate the anti-proliferation ability of compound **5i**. The number of cell clones was decreased when treated with compound **5i**, particularly in the 16 μM and 32 μM concentration groups in H460 cells (Figure 3C). Similarly, a reduction in the number of cell clones was also observed in H1299 cells following treatment with compound **5i** (Figure 3D). These results indicated that compound **5i** effectively inhibited cancer cell proliferation, thus underscoring its anticancer activity.

#### 3.2.3 Cell apoptosis investigation

**FIGURE 4:**
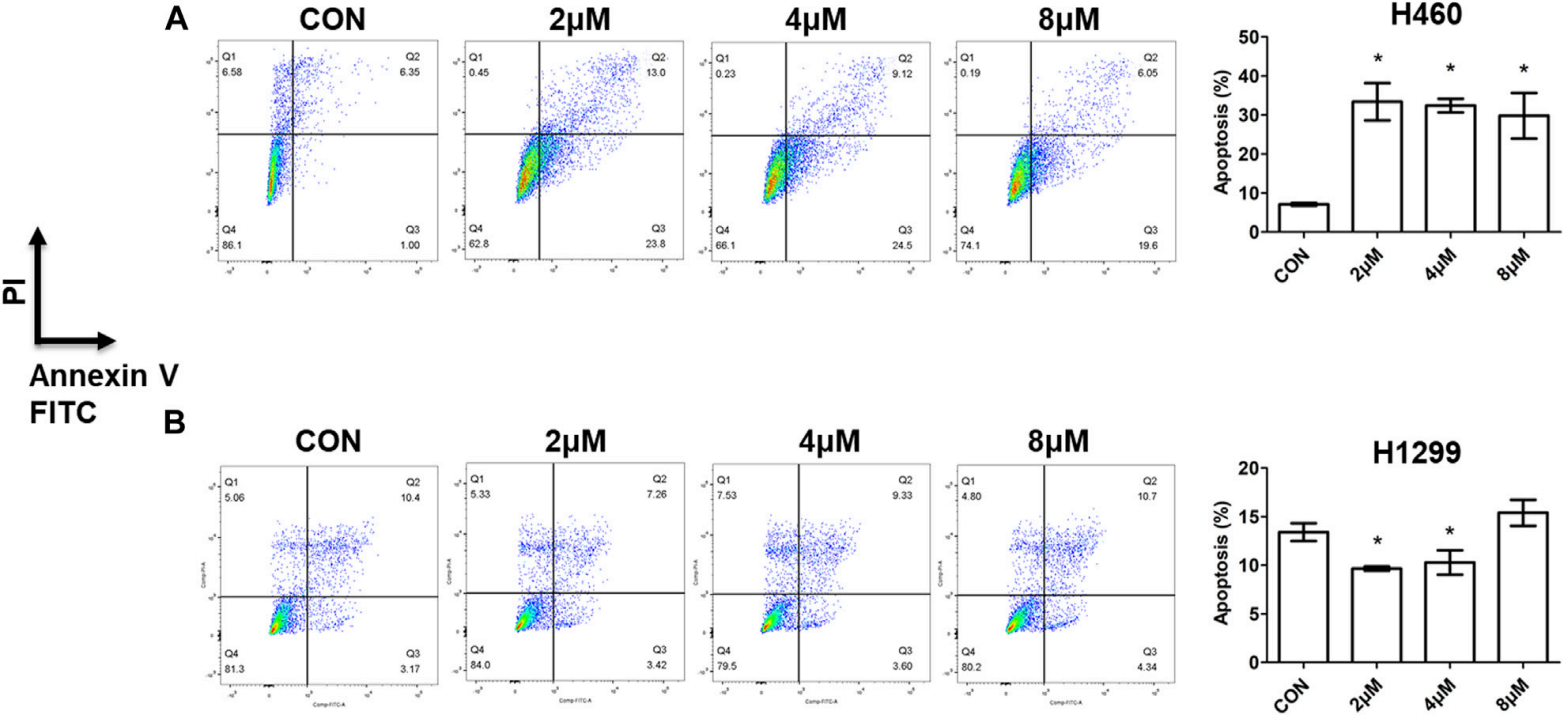
Cell apoptosis assay by flow cytometry of compound **5i** with different concentrations in H460 and H1299 cells. **(A)** Apoptotic cells detection in H460 cells; **(B)** Apoptotic cells detection in H1299 cells. Left: dot plot diagram; right, statistical chart. Data are presented as Mean ± SE. *p < 0.05.

Apoptosis is a programmed-controlled process of cell self-destruction, and increased cell apoptosis can lead to cell death (Wang et al., 2023). To explore the preliminary molecular mechanisms of the synthesized compounds action, the apoptotic activity of compound **5i** was evaluated by comparing the percentage of apoptotic cells with untreated cells using flow cytometry after staining with Annexin V-FITC and PI (Chen et al., 2022a). This assay facilitates the detection of necrotic cells (Q1, AV-/PI+), late apoptotic cells (Q2, AV+/PI+), early apoptotic cells (Q3, AV+/PI-), and live cells (Q4, AV-/PI-). H460 and H1299 cells were treated with different doses of compound **5i** for 72h, and cells were collected, stained, and analyzed. The proportion of early and late apoptotic cells was added and their ratio relative to the total cells was calculated. The results demonstrated a significant increase in apoptotic cells in H460 cells treated with 2, 4 and 8 μM of compound **5i** (Figure 4A). However, compound **5i** exhibited weaker impact on cell apoptosis in H1299 cells, as the apoptotic cell population showed no significant change or even a decrease (Figure 4B). This was consistent with the results of cell viability measurement that compound **5i** showed better antitumor effect on H460 cells rather than H1299 cells.

#### 3.2.4 ROS generation assay

**FIGURE 5:**
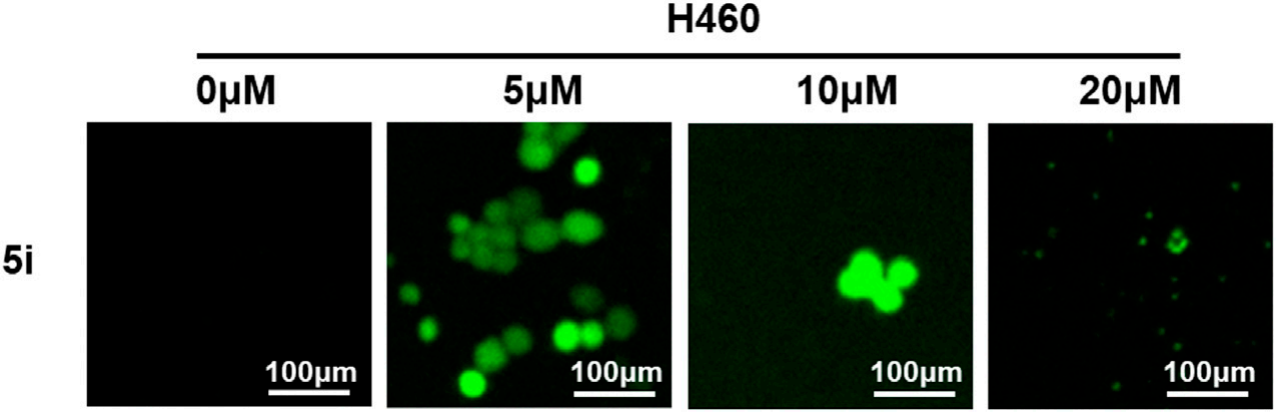
ROS generation detection of compound **5i** with different concentrations (5, 10, and 20 μM) in H460 cells.

Reactive oxygen species (ROS) levels were determined quantitatively using the probe 2′-7′-dichlorodihydrofluorescin diacetate (H2DCFDA) (Kim and Xue, 2020). Under normal condition, ROS participated in mitochondrial electron transport, signal transduction, gene expression, and enzyme reactions. But excess or deficiency of ROS levels can result in the development of diseases including cancers, diabetes, atherosclerosis, neurodegenerative diseases and others (Wang et al., 2023). ROS induction is associated with mitochondrial membrane damage, cell apoptosis, and ultimately cell death (Dixon and Stockwell, 2014). To evaluate the effect of compound **5i** on ROS generation, H460 cells were treated with 5 μM, 10 μM or 20 μM of compound **5i** for 24 h. Subsequently, the cells were stained with peroxide sensitive fluorescent dye, 2′-7′-dichlorodihydro-flurescien diacetate (DCFH-DA), and visualized using a fluorescent microscope. DCFH-DA has no fluorescence and it can pass through cell membrane freely. After entry the cell, it could hydrolyze by intracellular esterase to DCHF and intracellular ROS can further oxidize DCFH to produce green fluorescent DCF. Results showed a significant augmentation in ROS generation within H460 cells treated with compound **5i** (Figure 5). It is important to acknowledge that in the 10 μM and 20 μM groups, the ROS levels seemed less pronounced compared to the 5 μM group. This phenomenon can be attributed to the reduction in the number of live cells; however, the observed fluorescence appeared more intense (Figure 5). Previous studies have demonstrated that ROS production triggered apoptosis, autophagy, and cell death (Pellegrini et al., 2023). Our findings also showed that compound **5i** could increase ROS levels in H460 cells which may lead to cancer cell apoptosis and cell death.

#### 3.2.5 Western blot analysis

**FIGURE 6:**
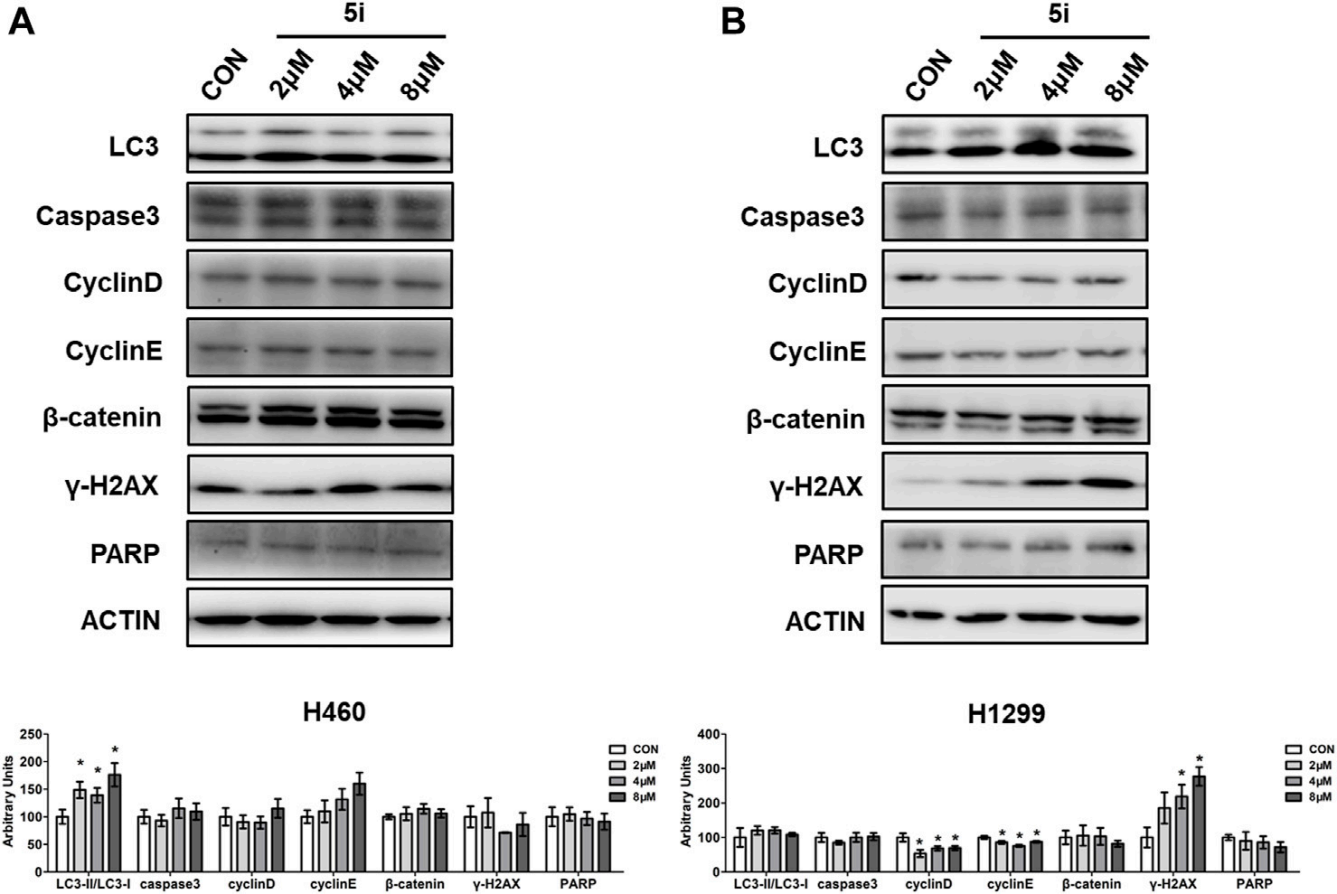
Western blot analysis of compound **5i** with different concentrations in H460 and H1299 cells. **(A)** Western blotting of LC3, Cyclin D, Cyclin E, β-catenin, γ-H2AX in H460 cells; **(B)** Western blotting of LC3, Cyclin D, Cyclin E, β-catenin, γ-H2AX in H1299 cells. Top, Western blot; bottom, quantitative measurements relative to ACTIN. The blots were cropped from different parts of the same gel or different gels, and the blots were processed in parallel. The full length original blot with edges visible was included in the Supporting Material. Data are presented as Mean ± SEM. *p < 0.05.

Western blot analysis was performed to assess the protein expressions involved in cell growth and cell death processes. H460 and H1299 cells were treated with different concentrations of compound **5i**, and whole cell proteins were collected for analysis. Results demonstrated a significant increase in the expression of ubiquitin like molecule light chain 3 (LC3) (Tanida et al., 2004), an important marker in autophagy, in H460 cells treated with compound **5i** (Figure 6A). This suggested that compound **5i** could induce autophagy. However, there were no significant differences observed in the expression of other proteins involved in cell cycle, DNA damage or DNA repair, such as caspase3, Cyclin D, Cyclin E, cadherin-associated protein beta 1 (β-catenin), γ-H2AX (Sharma et al., 2012), and Poly (ADP-Ribose) Polymerase (PARP) (Hanzlikova et al., 2018), after compound **5i** treatment in H460 cells (Figure 6A). In H1299 cells, no significant differences were observed in LC3 and caspase 3 expression levels (Figure 6B). Additionally, the levels of proteins related to cell cycle, including Cyclin D and Cyclin E, exhibited a decrease following treatment with compound **5i** (Figure 6B). Notably, β-catenin expression remained unaltered in response to the treatment (Figure 6B). Treatment with compound **5i** prompted DNA damage, as demonstrated by the increased expression of γ-H2AX, although PARP expression remained unaffected (Figure 6B). These results were consistent with the above. Autophagy has both tumor suppression and growth promotion effects in cancers, depending on the cellular context and cancer stage. On the one hand, autophagy inhibits cancer development via eliminating dysfunctional proteins and damaged mitochondria. On the other hand, autophagy can promote tumor growth by providing recycled metabolites to support cancer cell survival and growth (Wang et al., 2023). A number of studies have suggested that ROS induced autophagy as a regulator (Pellegrini et al., 2023). In our study, we found that compound **5i** significantly induced autophagy which was consistent with the stimulated ROS levels shown above. Combined with the above results, we suggested that compound **5i** promoted autophagy process and finally induced cell death in H460 cells. But in H1299 cells, compound **5i** exerted its anticancer ability by suppressing cell division and proliferation.

## 4 Conclusion

This study successfully synthesized a series of novel compounds by incorporating 1,2,3-triazole units into the cabotegravir backbone. The biological evaluations demonstrated that several of these molecular hybrids exhibited potent antitumor activities. Notably, the synthesized compounds displayed stronger cytotoxic effects against H460 cells than H1299 cells, with some compounds achieving cell viability rates below 50% in H460 cells but below 70% in H1299 cells. The effects became more pronounced with an extended duration of treatment. Compounds 5c, 5d, 5i, 5s, 6b, and 6 h showed a cell proliferation inhibition rate exceed 50% against H460 cell lines after 72-h treatment. Among them, compound **5i** emerged as the most potent cytotoxic agent, with an IC_50_ value of 6.06 μM. Besides, compounds **5c** and **5s** showed IC_50_ values of 9.65 μM and 13.38 μM, exhibited potential antitumor activities. Further studies found that compound **5i** significantly induced cell apoptosis and stimulated ROS generation, which may finally lead to cell death. Western blot analysis revealed the dysregulation of proteins associated with autophagy and DNA damage in either H460 or H1299 cells. These findings suggested that the novel derivatives especially compound **5i** holds promise as a potential therapeutic candidate for the treatment of lung cancer, warranting further investigations and explorations. 1,2,3-triazole derivatives have demonstrated potential antitumor activities against various types of tumors, which could be further investigated in our future work. Previous studies have also highlighted diverse activities of 1,2,3-triazoles, including antioxidant, antiviral, and acaricidal properties. In line with our findings, this novel compound induced ROS generation, indicating a potential antioxidant effect. Moving forward, our ongoing investigations will explore additional activities of these novel compounds.

## Data Availability

The original contributions presented in the study are included in the article/Supplementary Materials, further inquiries can be directed to the corresponding authors.
