# Supplementary material for: Synthesis and biological evaluation of novel 1,2,3-triazole hybrids of cabotegravir: identification of potent antitumor activity against lung cancer
Source: Front Pharmacol. 2023 Sep 20;14:1265245. doi: 10.3389/fphar.2023.1265245 (PMC10547880; doi:10.3389/fphar.2023.1265245)

Compound **5a**: yield 81.6%, white solid, HR-MS(ESI): Calcd. C28H23F3N6O5 [M+H]^+^ *m/z*: 581.1760, found: 581.1789. m.p. 177-180 ^o^C. ^1^H NMR(400MHz, DMSO-d_6_): 12.60 (d, J=8.0Hz, 1H), 9.10 (s, 1H), 8.72 (s, 1H), 8.25 (s, 1H), 8.08 (d, J=8.0Hz, 1H), 7.98 (t, J_1_=8.0Hz, J_2_=8.0Hz, 1H), 7.92-7.82 (m, 3H), 7.69 (d, J=8.0Hz, 1H), 7.50 (t, J_1_=8.0Hz, J_2_=8.0Hz, 1H), 5.54-5.34 (m, 1H), 4.98-4.79 (m, 1H), 4.46-4.36 (m, 1H), 4.19-4.05 (m, 2H), 3.92 (d, J=4.0Hz, 3H), 3.25-3.04 (m, 1H), 1.37-1.30 (m, 3H); ^13^C NMR(100MHz, DMSO-d_6_): 174.20, 162.27, 154.50, 154.34, 152.06, 147.75, 144.21, 139.51, 137.59, 131.86, 131.32, 130.36, 125.68, 125.46, 124.35, 121.34, 120.68, 120.18, 118.15, 117.08, 116.92, 82.74, 82.27, 74.75, 74.16, 61.00, 55.49, 54.96, 49.95, 18.41.

Compound **5b**: yield 87.1%, white solid, HR-MS(ESI): Calcd. C27H23FN6O5 [M+H]^+^ *m/z*: 531.1792, found: 531.1825. m.p. 199-202 ^o^C. ^1^H NMR(400MHz, DMSO-d_6_): 12.59 (s, 1H), 9.16 (s, 1H), 8.71 (s, 1H), 8.23 (s, 1H), 7.92 (t, J_1_=8.0Hz, J_2_=4.0Hz, 1H), 7.85 (d, J=4.0Hz, 1H), 7.71 (d, J=4.0Hz, 1H), 7.66-7.60 (m, 2H), 7.50 (dd, J_1_=4.0Hz, J_2_=8.0Hz, 2H), 5.54-5.34 (m, 1H), 4.96-4.79 (m, 1H), 4.46-4.36 (m, 2H), 3.92 (d, J=4.0Hz, 3H), 3.25-3.04 (m, 1H), 1.37-1.30 (m, 3H)；^13^C NMR(100MHz, DMSO-d_6_): 174.11, 162.27, 154.34, 153.09, 152.02, 147.11, 144.24, 144.13, 139.47, 131.85, 131.63, 131.32, 126.50, 126.11, 123.63, 121.46, 120.10, 118.14, 117.79, 116.97, 82.74, 74.15, 60.99, 55.45, 49.95, 18.14.

Compound **5c**: yield 77.4%, gray solid, HR-MS(ESI): Calcd. C28H27N6O5 [M+H]^+^ *m/z*: 527.2043, found: 527.2053. m.p. 188-191 ^o^C. ^1^H NMR(400MHz, DMSO-d_6_): 12.59 (s, 1H), 9.02 (s, 1H), 8.71 (s, 1H), 8.21 (s, 1H), 7.84 (d, J=4.0Hz, 1H), 7.69 (d, J=4.0Hz, 1H), 7.54-7.44 (m, 4H), 5.53-5.34 (m, 1H), 4.97-4.79 (m, 1H), 4.45-4.36 (m, 1H), 4.18-4.05 (m, 2H), 3.91 (d, J=4.0Hz, 3H), 3.25-3.04 (m, 1H), 1.37-1.30 (m, 3H). ^13^C NMR(100MHz, DMSO-d_6_):174.17, 162.25, 154.51, 152.02, 146.65, 144.12, 139.43, 136.75, 133.48, 131.91, 130.26, 127.52, 126.46, 123.81, 121.42, 119.92, 118.15, 116.89, 82.74, 82.27, 74.74, 74.15, 60.99, 55.37, 54.81, 49.55, 18.39, 17.99.

Compound **5d**: yield 65.8%, white solid, HR-MS(ESI): Calcd. C29H28N6O5 [M+H]^+^ *m/z*: 541.2199, found: 541.2210. m.p. 170-173 ^o^C. ^1^H NMR(400MHz, DMSO-d_6_): 12.60 (s, 1H), 9.02 (s, 1H), 8.72 (s, 1H), 8.22 (s, 1H), 7.84 (d, J=8.0Hz, 1H), 7.69 (d, J=4.0Hz, 1H), 7.59-7.45 (m, 5H), 5.54-5.34 (m, 1H), 4.98-4.80 (m, 1H), 4.46-4.36 (m, 1H), 4.19-4.04 (m, 2H), 3.91 (d, J=4.0Hz, 3H), 3.25-3.04 (m, 1H), 2.56-2.52 (m, 2H), 1.37-1.30 (m, 3H), 1.07 (t, J_1_=8.0Hz, J_2_=8.0Hz, 3H). ^13^C NMR(100MHz, DMSO-d_6_):174.10, 162.23, 154.32, 151.99, 146.65, 144.21, 139.75, 136.21, 131.69, 130.73, 130.37, 130.25, 127.47, 126.89, 124.11, 121.40, 119.92, 118.03, 116.88, 82.73, 82.27, 74.73, 74.14, 60.97, 55.43, 49.94, 24.30, 18.38, 15.33.

Compound **5e**: yield 61.1%, white solid, HR-MS(ESI): Calcd. C29H22F6N6O5 [M+H]^+^ *m/z*: 649.1634, found: 649.1650. m.p. 274-277 ^o^C. ^1^H NMR(400MHz, DMSO-d_6_): 12.63 (d, J=4.0Hz, 1H), 9.67 (s, 1H), 8.69 (s, 1H), 8.29 (s, 1H), 8.15 (s, 1H), 7.86 (d, J=8.0Hz, 1H), 7.68 (d, J=8.0Hz, 1H), 7.51 (t, J_1_=8.0Hz, J_2_=8.0Hz, 1H), 5.55-5.34 (m, 1H), 4.98-4.79 (m, 1H), 4.47-4.36 (m, 1H), 4.19-4.05 (m, 2H), 3.92-3.91 (m, 3H), 3.25-3.04 (m, 1H), 1.38-1.31 (m, 3H). ^13^C NMR(100MHz, DMSO-d_6_): 174.15, 162.23, 154.31, 151.96, 147.89, 144.18, 139.51, 138.31, 132.50, 132.16, 130.99, 130.41, 124.64, 121.92, 120.93, 120.23, 118.10, 116.77, 82.72, 82.26, 74.74, 74.15, 60.98, 55.43, 54.80, 49.94, 18.39.

Compound **5f**: yield 72.5%, white solid, HR-MS(ESI): Calcd. C27H23FN6O5 [M+H]^+^ *m/z*: 531.1792, found: 531.1810. m.p. 211-214 ^o^C. ^1^H NMR(400MHz, DMSO-d_6_): 12.68 (s, 1H), 9.50 (s, 1H), 8.77 (s, 1H), 8.22 (s, 1H), 7.98-7.93 (m, 3H), 7.78-7.74 (m, 2H), 7.57 (t, J_1_=4.0Hz, J_2_=4.0Hz, 1H), 7.45 (t, J_1_=4.0Hz, J_2_=8.0Hz, 1H), 5.60-5.40 (m, 1H), 5.03-4.85 (m, 1H), 4.52-4.42 (m, 1H), 4.24-4.11 (m, 2H), 3.98-3.97 (m, 3H), 3.31-3.11 (m, 1H), 1.43-1.36 (m, 3H). ^13^C NMR(100MHz, DMSO-d_6_): 174.13, 164.16, 162.26, 154.50, 152.02, 147.61, 144.11, 139.50, 138.27, 132.45, 131.32, 130.37, 121.31, 120.56, 120.13, 118.13, 116.86, 116.34, 115.82, 108.07, 82.74, 82.27,74.74, 74.15, 60.99, 55.38, 18.39.

Compound **5g**: yield 86.6%, white solid, HR-MS(ESI): Calcd. C28H26N6O6 [M+H]^+^ *m/z*: 543.1992, found: 543.2007. m.p. 253-256 ^o^C. ^1^H NMR(400MHz, DMSO-d_6_): 12.59 (s, 1H), 9.00 (s, 1H), 8.74 (s, 1H), 8.23 (s, 1H), 7.84 (d, J=8.0Hz, 1H), 7.72-7.70 (m, 2H), 7.59 (t, J_1_=8.0Hz, J_2_=8.0Hz, 1H), 7.50 (t, J_1_=8.0Hz, J_2_=8.0Hz, 1H), 7.39-7.37 (m, 1H), 7.20 (t, J_1_=8.0Hz, J_2_=8.0Hz, 1H), 5.56-5.36 (m, 1H), 5.00-4.80 (m, 1H), 4.48-4.37 (m, 1H), 4.21-4.06 (m, 2H), 3.94-3.92 (m, 6H), 3.28-3.06 (m, 1H), 1.39-1.32 (m, 3H). ^13^C NMR(100MHz, DMSO-d_6_): 174.17, 162.25, 154.51, 152.31, 152.01, 146.39, 144.23, 139.42, 131.76, 131.39, 130.23, 126.43, 126.20, 124.14, 121.36, 119.17, 118.17, 116.86, 113.49, 82.74, 82.28, 74.74, 74.15, 60.99, 56.66, 55.44, 49.95, 18.39.

Compound **5h**: yield 90.1%, white solid, HR-MS(ESI): Calcd. C28H23F3N6O5 [M+H]^+^ *m/z*: 581.1760, found: 581.1776. m.p. 160-163 ^o^C. ^1^H NMR(400MHz, DMSO-d_6_): 12.64 (s, 1H), 9.58 (s, 1H), 8.73 (s, 1H), 8.39-8.36 (m, 1H), 8.21(s, 1H), 7.93-7.89 (m, 3H), 7.73 (d, J=8.0Hz, 1H), 7.54 (t, J_1_=8.0Hz, J_2_=8.0Hz, 1H), 5.57-5.36 (m, 1H), 5.00-4.81 (m, 1H), 4.49-4.38 (m, 1H), 4.20-4.19 (m, 1H), 4.12-4.09 (m, 1H), 3.94-3.93 (m, 3H), 3.28-3.06 (m, 1H), 1.40-1.32 (m, 3H). ^13^C NMR(100MHz, DMSO-d_6_):174.19, 162.27, 154.33, 152.02, 147.73, 144.11, 139.51, 137.58, 131.88, 131.31, 130.39, 125.74, 125.01, 124.34, 121.32, 120.71, 120.16, 118.00, 116.88, 82.28, 74.16, 67.40, 61.00, 55.46, 49.56, 49.07, 31.78, 18.40.

Compound **5i**: yield 72.2%, white solid, HR-MS(ESI): Calcd. C28H23F3N6O6 [M+H]^+^ *m/z*: 597.1709, found: 597.1713. m.p. 263-266 ^o^C. ^1^H NMR(400MHz, DMSO-d_6_): 12.62 (s, 1H), 9.17 (s, 1H), 8.74 (d, J=4.0Hz, 1H), 8.26 (s, 1H), 7.96-7.94 (m, 1H), 7.87-7.85 (m, 1H), 7.79-7.78 (m, 2H), 7.74-7.70 (m, 2H), 7.52 (t, J_1_=8.0Hz, J_2_=8.0Hz, 1H), 5.56-5.36 (m, 1H), 5.00-4.80 (m, 1H), 4.48-4.38 (m, 1H), 4.21-4.06 (m, 2H), 3.94-3.93 (m, 3H), 3.28-3.06 (m, 1H), 1.39-1.32 (m, 3H). ^13^C NMR(100MHz, DMSO-d_6_):174.18, 162.27, 154.50, 154.33, 152.01, 146.95, 144.24, 141.61, 139.50, 132.18, 131.64, 131.30, 130.35, 130.18, 129.36, 128.06, 123.96, 121.41, 120.12, 116.93, 82.74, 74.74, 60.99, 55.45, 49.95, 18.41.

Compound **5j**: yield 54.3%, white solid, HR-MS(ESI): Calcd. C28H23F3N6O5 [M+H]^+^ *m/z*: 581.1760, found: 581.1769. m.p. 216-219 ^o^C. ^1^H NMR(400MHz, DMSO-d_6_): 12.65 (s, 1H), 9.58 (s, 1H), 8.74 (d, J=4.0Hz, 1H), 8.29-8.22 (m, 3H), 8.07 (d, J=8.0Hz, 1H), 7.92-7.90 (m, 1H), 7.75-7.73 (m, 1H), 7.54 (t, J_1_=8.0Hz, J_2_=8.0Hz, 1H), 5.57-5.36 (m, 1H), 5.00-4.81 (m, 1H), 4.49-4.38 (m, 1H), 4.21-4.07 (m, 2H), 3.94-3.93 (m, 3H), 3.28-3.08 (m, 1H), 1.40-1.32 (m, 3H). ^13^C NMR(150MHz, DMSO-d_6_):173.12, 161.20, 153.26, 150.94, 146.74, 143.15, 138.43, 130.59, 130.16, 129.31, 126.71, 120.28, 119.79, 119.55, 119.12, 116.92, 115.83, 81.66, 81.19, 73.66, 73.07, 59.91, 54.38, 48.87, 17.31.

Compound **5k**: yield 66.9%, white solid, HR-MS(ESI): Calcd. C29H22F6N6O5 [M+H]^+^ *m/z*: 649.1634, found: 649.1679. m.p. 172-175 ^o^C. ^1^H NMR(400MHz, DMSO-d_6_): 12.63 (s, 1H), 9.20 (s, 1H), 8.73 (d, J=4.0Hz, 1H), 8.46 (s, 1H), 8.37-8.27 (m, 3H), 7.86-7.83 (m, 1H), 7.72-7.70 (m, 1H), 7.53 (t, J_1_=8.0Hz, J_2_=8.0Hz, 1H), 5.56-5.36 (m, 1H), 5.00-4.80 (m, 1H), 4.49-4.38 (m, 1H), 4.21-4.07 (m, 2H), 3.94-3.90 (m, 3H), 3.28-3.08 (m, 1H), 1.39-1.32 (m, 3H). ^13^C NMR(100MHz, DMSO-d_6_): 174.18, 162.28, 154.33, 152.02, 146.86, 144.11, 139.53, 135.62, 131.51, 130.40, 129.76, 128.69, 127.11, 125.17, 124.06, 121.39, 120.18, 118.00, 116.95, 82.74, 82.28, 74.74, 74.15, 60.98, 55.46, 54.82, 49.55, 18.39.

Compound **5l**: yield 56.9%, white solid, HR-MS(ESI): Calcd. C30H30N6O5 [M+H]^+^ *m/z*: 555.2356, found: 555.2397. m.p. 181-184 ^o^C. ^1^H NMR(400MHz, DMSO-d_6_): 12.59-12.57 (m, 1H), 8.85 (s, 1H), 8.71 (s, 1H), 8.21 (s, 1H), 7.82 (d, J=4.0Hz, 1H), 7.67 (d, J=8.0Hz, 1H), 7.48 (t, J_1_=8.0Hz, J_2_=8.0Hz, 1H), 7.13 (s, 2H), 5.53-5.34 (m, 1H), 4.96-4.79 (m, 1H), 4.45-4.36 (m, 1H), 4.18-4.12 (m, 1H), 4.09-4.05 (m, 1H), 3.91-3.90 (m, 3H), 3.25-3.04 (m, 1H), 2.35 (s, 3H), 1.96 (s, 6H), 1.37-1.30 (m, 3H). ^13^C NMR(100MHz, DMSO-d_6_): 174.11, 162.24, 154.34, 152.00, 146.61, 144.22, 140.07, 139.43, 134.96, 133.87, 131.83, 130.24, 129.41, 124.19, 121.39, 119.87, 118.03, 116.88, 82.74, 74.74, 74.15, 60.98, 55.45, 49.95, 21.14, 18.42, 17.37.

Compound **5m**: yield 77.2%, white solid, HR-MS(ESI): Calcd. C27H23BrN6O5 [M+H]^+^ *m/z*: 591.0992, found: 591.1010. m.p. 207-210 ^o^C. ^1^H NMR(400MHz, DMSO-d_6_): 12.60-12.59 (m, 1H), 9.10 (s, 1H), 8.71 (s, 1H), 8.23 (s, 1H), 8.00-7.95 (m, 1H), 7.89-7.82 (m, 1H), 7.77-7.48 (m, 5H), 5.54-5.34 (m, 1H), 4.98-4.78 (m, 1H), 4.48-4.34 (m, 1H), 4.19-4.04 (m, 2H), 3.91-3.90 (m, 3H), 3.25-3.03 (m, 1H), 1.37-1.30 (m, 3H). ^13^C NMR(100MHz, DMSO-d_6_): 174.18, 162.26, 154.34, 152.03, 146.64, 144.23, 139.47, 136.68, 134.15, 132.56, 131.50, 130.31, 129.48, 129.18, 124.39, 121.40, 120.44, 120.02, 119.37, 118.03, 116.90, 82.74, 82.27, 74.74, 74.15, 60.98, 56.50, 49.94, 19.01.

Compound **5n**: yield 65.5%, white solid, HR-MS(ESI): Calcd. C27H23FN6O5 [M+H]^+^ *m/z*: 531.1792, found: 531.1821. m.p. 277-280 ^o^C. ^1^H NMR(400MHz, DMSO-d_6_): 12.61 (d, J=4.0Hz, 1H), 9.36 (s, 1H), 8.71 (s, 1H), 8.17 (s, 1H), 8.06-8.02 (m, 1H), 7.87 (d, J=8.0Hz, 1H), 7.70 (d, J=8.0Hz, 1H), 7.51 (t, J_1_=8.0Hz, J_2_=8.0Hz, 3H), 5.55-5.35 (m, 1H), 4.98-4.79 (m, 1H), 4.47-4.36 (m, 1H), 4.21-4.05 (m, 2H), 3.92 (d, J=4.0Hz, 3H), 3.27-3.05 (m, 1H), 1.38-1.31 (m, 3H). ^13^C NMR(100MHz, DMSO-d_6_): 174.14, 162.26, 160.95, 154.34, 152.06, 147.53, 144.10, 139.48, 133.73, 131.63, 131.51, 130.31, 122.87, 121.34, 120.63, 120.06, 118.17, 117.38, 117.15, 116.90, 82.76, 74.16, 61.00, 55.49, 54.85, 49.56, 18.41.

Compound **5o**: yield 42.7%, white solid, HR-MS(ESI): Calcd. C27H23IN6O5 [M+H]^+^ *m/z*: 639.0853, found: 639.0868. m.p. 181-184 ^o^C. ^1^H NMR(400MHz, DMSO-d_6_): 12.61 (d, J=4.0Hz, 1H), 9.40 (s, 1H), 8.72 (s, 1H), 8.18 (s, 1H), 8.00-7.98 (m, 1H), 7.88 (d, J=8.0Hz, 1H), 7.72-7.63 (m, 3H), 7.55-7.49 (m, 3H), 5.55-5.34 (m, 1H), 4.98-4.79 (m, 1H), 4.46-4.35 (m, 1H), 4.19-4.05 (m, 2H), 3.92-3.91 (m, 3H), 3.25-3.04 (m, 1H), 1.37-1.30 (m, 3H). ^13^C NMR(100MHz, DMSO-d_6_): 174.18, 162.25, 154.50, 151.99, 147.49, 144.22, 144.11, 139.47, 137.12, 131.54, 130.42, 130.31, 129.21, 121.32, 120.44, 120.36, 120.01, 118.02, 116.85, 99.99, 82.27, 74.15, 60.99, 54.80, 49.55, 18.41.

Compound **5p**: yield 68.4%, white solid, HR-MS(ESI): Calcd. C27H24N6O5 [M+H]^+^ *m/z*: 513.1886, found: 513.1895. m.p. 229-232 ^o^C. ^1^H NMR(400MHz, DMSO-d_6_): 12.63 (d, J=4.0Hz, 1H), 9.41 (s, 1H), 8.73 (d, J=4.0Hz,1H), 8.18 (s, 1H), 8.01-7.98 (m, 1H), 7.88 (d, J=12.0Hz, 1H), 7.72-7.63 (m, 3H), 7.55-7.49 (m, 3H), 5.55-5.34 (m, 1H), 4.98-4.79 (m, 1H), 4.48-4.36 (m, 1H), 4.20-4.05 (m, 2H), 3.92 (d, J=4.0Hz, 3H), 3.26-3.04 (m, 1H), 1.37-1.30 (m, 3H). ^13^C NMR(100MHz, DMSO-d_6_):174.18, 162.26, 154.34, 152.00, 147.49, 144.13, 139.46, 137.11, 131.66, 131.54, 130.43, 130.33, 129.23, 121.34, 120.44, 118.13, 116.84, 82.73, 82.27, 74.74, 74.15, 60.99, 55.44, 49.55, 18.39.

Compound **5q**: yield 63.9%, white solid, HR-MS(ESI): Calcd. C27H23ClN6O5 [M+H]^+^ *m/z*: 547.1497, found: 547.1510. m.p. 204-207 ^o^C. ^1^H NMR(600MHz, DMSO-d_6_): 12.62 (d, J=6.0Hz, 1H), 9.47 (s, 1H), 8.71 (d, J=6.0Hz, 1H), 8.16 (s, 1H), 8.12 (t, J_1_=6.0Hz, J_2_=6.0Hz, 1H), 8.02-8.00 (m, 1H), 7.88 (d, J=12.0Hz, 1H), 7.70-7.66 (m, 2H), 7.60 (d, J=12.0Hz, 1H), 7.51 (t, J_1_=6.0Hz, J_2_=6.0Hz, 1H), 5.54-5.34 (m, 1H), 4.97-4.79 (m, 1H), 4.46-4.37 (m, 1H), 4.18-4.05 (m, 2H), 3.91(d, J=6.0Hz, 3H), 3.25-3.05 (m, 1H), 1.37-1.30 (m, 3H). ^13^C NMR(150MHz, DMSO-d_6_): 174.19, 162.26, 154.50, 154.33, 152.00, 147.62, 144.21, 144.11, 139.50, 138.15, 134.73, 132.18, 131.65, 131.53, 131.33, 130.37, 128.99, 121.30, 120.55, 120.22, 119.01, 118.01, 116.85, 82.74, 74.75, 74.15, 60.99, 55.46, 49.95, 18.40.

Compound **5r**: yield 86.2%, white solid, HR-MS(ESI): Calcd. C27H23BrN6O5 [M+H]^+^ *m/z*: 591.0992, found: 591.0998. m.p. 231-234 ^o^C. ^1^H NMR(600MHz, DMSO-d_6_): 12.62 (d, J=6.0Hz, 1H), 9.46 (s, 1H), 8.71 (d, J=6.0Hz, 1H), 8.24 (s, 1H), 8.16 (s, 1H), 8.05-8.04 (m, 1H), 7.88 (d, J=12.0Hz, 1H), 7.74-7.68 (m, 2H), 7.60 (t, J_1_=12.0Hz, J_2_=6.0Hz, 1H), 7.51 (t, J_1_=6.0Hz, J_2_=12.0Hz, 1H), 5.54-5.34 (m, 1H), 4.97-4.79 (m, 1H), 4.46-4.37 (m, 1H), 4.18-4.05 (m, 2H), 3.92 (d, J=6.0Hz, 3H), 3.25-3.04 (m, 1H), 1.37-1.30 (m, 3H). ^13^C NMR(150MHz, DMSO-d_6_): 174.19, 162.25, 154.50, 154.33, 152.02, 147.60, 144.10, 139.50, 138.22, 132.38, 131.90, 131.51, 131.33, 130.35, 122.95, 121.29, 120.53, 120.11, 119.38, 118.14, 116.84, 82.28, 74.15, 60.99, 54.82, 49.56, 18.40.

Compound **5s**: yield 80.8%, white solid, HR-MS(ESI): Calcd. C27H23ClN6O5 [M+H]^+^ *m/z*: 547.1497, found: 547.1511. m.p. 259-262 ^o^C. ^1^H NMR(600MHz, DMSO-d_6_): 12.60 (d, J=6.0Hz, 1H), 9.12 (s, 1H), 8.72 (d, J=6.0Hz, 1H), 8.23 (s, 1H), 7.84-7.79 (m, 3H), 7.69-7.62 (m, 3H), 7.50 (t, J_1_=6.0Hz, J_2_=12.0Hz, 1H), 5.54-5.34 (m, 1H), 4.97-4.79 (m, 1H), 4.45-4.36 (m, 1H), 4.18-4.05 (m, 2H), 3.91 (d, J=6.0Hz, 3H), 3.25-3.04 (m, 1H), 1.37-1.30 (m, 3H). ^13^C NMR(150MHz, DMSO-d_6_): 174.18, 162.27, 154.34, 152.01, 146.70, 144.23, 139.48, 134.99, 132.27, 131.63, 131.44, 131.10, 130.32, 129.01, 128.92, 124.39, 121.42, 120.05, 118.15, 116.92, 82.74, 74.74, 74.15, 60.99, 55.45, 49.95, 18.42.

Compound **6a**: yield 76.8%, white solid, HR-MS(ESI): Calcd. C28H26N6O5 [M+H]^+^ *m/z*: 527.2043, found: 527.2031. m.p. 260-263 ^o^C. ^1^H NMR(600MHz, DMSO-d_6_): 12.55 (d, J=6.0Hz, 1H), 8.71 (s, 1H), 8.71 (d, J=6.0Hz, 1H), 8.13 (s, 1H), 7.76 (d, J=6.0Hz, 1H), 7.59 (d, J=6.0Hz, 1H), 7.45-7.34 (m, 6H), 5.65 (s, 2H), 5.53-5.33 (m, 1H), 4.96-4.77 (m, 1H), 4.45-4.36 (m, 1H), 4.18-4.05 (m, 2H), 3.91 (d, J=6.0Hz, 3H), 3.25-3.04 (m, 1H), 1.36-1.30 (m, 3H). ^13^C NMR(150MHz, DMSO-d_6_): 174.16, 168.40, 162.21, 154.34, 151.99, 146.84, 144.20, 139.36, 136.46, 131.95, 131.62, 130.19, 129.30, 128.67, 128.45, 122.33, 121.18, 119.67, 118.03, 116.71, 82.74, 74.14, 60.97, 55.44, 53.55, 49.94, 18.39.

Compound **6b**: yield 72.4%, white solid, HR-MS(ESI): Calcd. C28H25BrN6O5 [M+H]^+^ *m/z*: 605.1148, found: 605.1155. m.p. 259-262 ^o^C. ^1^H NMR(600MHz, DMSO-d_6_): 12.55 (d, J=6.0Hz, 1H), 8.70 (d, J=6.0Hz, 1H), 8.67 (s, 1H), 8.14 (s, 1H), 7.76 (d, J=12.0Hz, 1H), 7.72 (d, J=6.0Hz, 1H), 7.61 (d, J=6.0Hz, 1H), 7.44 (t, J_1_=6.0Hz, J_2_=6.0Hz, 2H), 7.34 (t, J_1_=6.0Hz, J_2_=6.0Hz, 1H), 7.27 (d, J=6.0Hz, 1H), 5.75 (s, 2H), 5.53-5.34 (m, 1H), 4.96-4.78 (m, 1H), 4.45-4.36 (m, 1H), 4.17-4.04 (m, 2H), 3.91 (d, J=6.0Hz, 3H), 3.24-3.04 (m, 1H), 1.36-1.30 (m, 3H). ^13^C NMR(150MHz, DMSO-d_6_): 174.09, 162.22, 154.34, 152.02, 146.66, 144.10, 139.37, 135.24, 133.42, 131.86, 131.06, 130.95, 130.19, 128.83, 123.39, 122.72, 121.25, 119.73, 118.16, 116.77, 82.27, 74.14, 60.98, 54.80, 53.63, 49.55, 18.39.

Compound **6c**: yield 85.2%, white solid, HR-MS(ESI): Calcd. C29H28N6O5 [M+H]^+^ *m/z*: 541.2199, found: 541.2241. m.p. 272-275 ^o^C. ^1^H NMR(600MHz, DMSO-d_6_): 12.54 (d, J=6.0Hz, 1H), 8.70 (d, J=6.0Hz, 1H), 8.60 (s, 1H), 8.13 (s, 1H), 7.76 (d, J=12.0Hz, 1H), 7.60 (d, J=6.0Hz, 1H), 7.43 (t, J_1_=6.0Hz, J_2_=6.0Hz, 1H), 7.28-7.16 (m, 4H), 5.66 (s, 2H), 5.53-5.34 (m, 1H), 4.96-4.77 (m, 1H), 4.47-4.36 (m, 1H), 4.18-4.04 (m, 2H), 3.91 (d, J=6.0Hz, 3H), 3.24-3.04 (m, 1H), 2.36 (s, 3H), 1.36-1.30 (m, 3H). ^13^C NMR(150MHz, DMSO-d_6_):174.15, 162.20, 154.33, 151.99, 146.71, 144.09, 139.35, 136.83, 134.51, 131.94, 130.93, 130.16, 129.26, 128.85, 126.80, 122.31, 121.20, 119.16, 118.16, 116.74, 82.73, 74.14, 60.97, 54.80, 51.65, 49.55, 31.78, 19.18, 18.39.

Compound **6d**: yield 88.6%, white solid, HR-MS(ESI): Calcd. C29H25F3N6O5 [M+H]^+^ *m/z*: 595.1917, found: 595.1928. m.p. 251-254 ^o^C. ^1^H NMR(400MHz, DMSO-d_6_): 12.56 (d, J=4.0Hz, 1H), 8.75 (s, 1H), 8.70 (d, J=4.0Hz, 1H), 8.14 (s, 1H), 7.78 (t, J_1_=4.0Hz, J_2_=8.0Hz, 3H), 7.60-7.56 (m, 3H), 7.44 (t, J_1_=8.0Hz, J_2_=4.0Hz, 1H), 5.79 (s, 2H), 5.53-5.34 (m, 1H), 4.96-4.78 (m, 1H), 4.45-4.36 (m, 1H), 4.18-4.05 (m, 2H), 3.91-3.90 (m, 3H), 3.24-3.04 (m, 1H), 1.37-1.30 (m, 3H). ^13^C NMR(100MHz, DMSO-d_6_): 174.16, 162.21, 154.33, 151.99, 146.96, 144.19, 144.08, 141.10, 139.38, 131.85, 131.62, 130.21, 129.15, 126.21, 122.63, 121.20, 119.73, 118.15, 118.03, 116.75, 82.74, 74.74, 60.97, 55.45, 52.88, 49.94, 18.39.

Compound **6e**: yield 59.3%, white solid, HR-MS(ESI): Calcd. C29H25F3N6O5 [M+H]^+^ *m/z*: 595.1917, found: 595.1943. m.p. 267-270 ^o^C. ^1^H NMR(600MHz, DMSO-d_6_): 12.55 (d, J=6.0Hz, 1H), 8.70 (d, J=6.0Hz, 2H), 8.15 (s, 1H), 7.85 (d, J=12.0Hz, 1H), 7.77 (d, J=6.0Hz, 1H), 7.72 (t, J_1_=6.0Hz, J_2_=6.0Hz, 1H), 7.61 (t, J_1_=6.0Hz, J_2_=12.0Hz, 2H), 7.44 (t, J_1_=12.0Hz, J_2_=6.0Hz, 1H), 7.28 (d, J=6.0Hz, 1H), 5.86 (s, 2H), 5.53-5.34 (m, 1H), 4.96-4.78 (m, 1H), 4.45-4.36 (m, 1H), 4.18-4.05 (m, 2H), 3.91 (d, J=6.0Hz, 3H), 3.24-3.04 (m, 1H), 1.36-1.30 (m, 3H). ^13^C NMR(150MHz, DMSO-d_6_): 174.09, 162.22, 154.33, 151.99, 146.80, 144.20, 139.38, 134.05, 133.75, 131.81, 131.62, 131.49, 130.80, 130.19, 129.43, 127.19, 126.74, 125.56, 122.92, 121.25, 119.76, 118.15, 116.79, 82.74, 74.14, 60.97, 55.44, 50.25, 49.55, 18.41.

Compound **6f**: yield 67.2%, white solid, HR-MS(ESI): Calcd. C28H25ClN6O5 [M+H]^+^ *m/z*: 561.1653, found: 561.1699. m.p. 262-265 ^o^C. ^1^H NMR(600MHz, DMSO-d_6_): 12.55 (d, J=6.0Hz, 1H), 8.70-8.69 (m, 2H), 8.12 (s, 1H), 7.76 (d, J=12.0Hz, 1H), 7.57 (d, J=6.0Hz, 1H), 7.48-7.40 (m, 5H), 5.66 (s, 2H), 5.53-5.34 (m, 1H), 4.96-4.77 (m, 1H), 4.46-4.36 (m, 1H), 4.18-4.05 (m, 2H), 3.91 (d, J=6.0Hz, 3H), 3.24-3.04 (m, 1H), 1.37-1.30 (m, 3H). ^13^C NMR(150MHz, DMSO-d_6_): 174.16, 162.22, 154.52, 152.02, 146.88, 144.20, 139.37, 135.42, 133.39, 131.89, 131.62, 130.43, 130.20, 129.30, 122.38, 121.18, 119.70, 118.16, 116.73, 82.74, 74.74, 60.98, 52.74, 49.55, 18.42.

Compound **6g**: yield 49.4%, white solid, HR-MS(ESI): Calcd. C29H28N6O6 [M+H]^+^ *m/z*: 557.2149, found: 557.2148. m.p. 251-254 ^o^C. ^1^H NMR(600MHz, DMSO-d_6_): 12.55 (d, J=6.0Hz, 1H), 8.70-8.69 (m, 2H), 8.13 (s, 1H), 7.76 (d, J=6.0Hz, 1H), 7.59 (d, J=6.0Hz, 1H), 7.43 (t, J_1_=12.0Hz, J_2_=6.0Hz, 1H), 7.31 (t, J_1_=12.0Hz, J_2_=6.0Hz, 1H), 6.97 (s, 1H), 6.93-6.91 (m, 2H), 5.62 (s, 2H), 5.53-5.34 (m, 1H), 4.96-4.77 (m, 1H), 4.45-4.36 (m, 1H), 4.19-4.05 (m, 2H), 3.91 (d, J=6.0Hz, 3H), 3.76 (s, 3H), 3.24-3.04 (m, 1H), 1.36-1.30 (m, 3H). ^13^C NMR(150MHz, DMSO-d_6_): 174.09, 162.21, 159.96, 154.34, 151.99, 146.82, 144.19, 139.36, 137.86, 131.95, 131.49, 130.46, 130.18, 122.33, 121.18, 120.53, 119.67, 118.16, 116.71, 114.27, 114.01, 82.74, 74.14, 60.97, 55.61, 53.48, 49.94, 18.39.

Compound **6h**: yield 77.1%, white solid, HR-MS(ESI): Calcd. C28H25ClN6O5 [M+H]^+^ *m/z*: 561.1653,found: 561.1657. m.p. 272-275 ^o^C. ^1^H NMR(600MHz, DMSO-d_6_): 12.55 (d, J=6.0Hz, 1H), 8.70 (d, J=6.0Hz, 1H), 8.68 (s, 1H), 8.14 (s, 1H), 7.76 (d, J=12.0Hz, 1H), 7.61 (d, J=6.0Hz, 1H), 7.55 (d, J=12.0Hz, 1H), 7.45-7.39 (m, 3H), 7.32 (d, J=12.0Hz, 1H), 5.77 (s, 2H), 5.53-5.33 (m, 1H), 4.96-4.78 (m, 1H), 4.45-4.36 (m, 1H), 4.17-4.04 (m, 2H), 3.91 (d, J=6.0Hz, 3H), 3.24-3.04 (m, 1H), 1.36-1.30 (m, 3H). ^13^C NMR(150MHz, DMSO-d_6_): 174.15, 162.21, 154.33, 152.01, 146.66, 144.20, 139.37, 133.63, 133.15, 131.86, 131.61, 131.10, 130.78, 130.14, 128.28, 122.68, 121.24, 119.72, 118.03, 116.76, 82.74, 74.14, 60.98, 55.44, 54.80, 51.30, 49.94, 18.39.

Compound **6i**: yield 65.1%, white solid, HR-MS(ESI): Calcd. C28H25FN6O5 [M+H]^+^ *m/z*: 545.1949, found: 545.1937. m.p. 275-278 ^o^C. ^1^H NMR(600MHz, DMSO-d_6_): 12.55 (d, J=12.0Hz, 1H), 8.74 (s, 1H), 8.70 (d, J=6.0Hz, 1H), 8.14 (s, 1H), 7.77 (d, J=12.0Hz, 1H), 7.60 (d, J=6.0Hz, 1H), 7.45-7.43 (m, 2H), 7.26-7.18 (m, 3H), 5.69 (s, 2H), 5.53-5.34 (m, 1H), 4.96-4.78 (m, 1H), 4.45-4.36 (m, 1H), 4.18-4.05 (m, 2H), 3.91 (d, J=6.0Hz, 3H), 3.24-3.04 (m, 1H), 1.36-1.30 (m, 3H). ^13^C NMR(150MHz, DMSO-d_6_): 174.16, 163.46, 162.21, 154.50, 152.02, 146.90, 144.19, 139.37, 139.04, 131.88, 131.37, 130.19, 124.56, 122.47, 121.19, 119.71, 118.03, 116.74, 115.62, 115.46, 115.31, 82.27, 74.14, 60.97, 55.45, 52.86, 49.94, 18.41.

# Figure S1. ^1^H NMR and ^13^C NMR spectrums of compound 5a


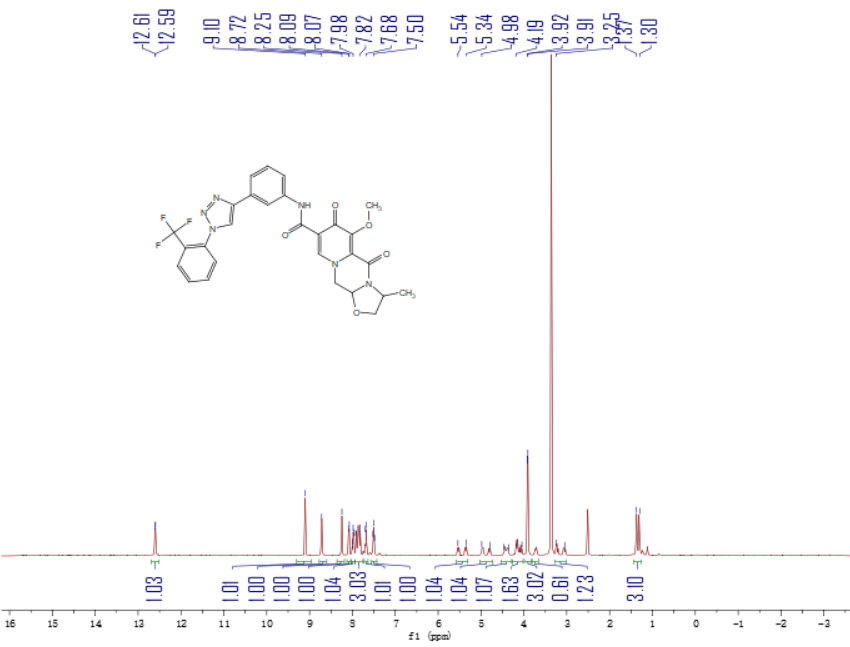


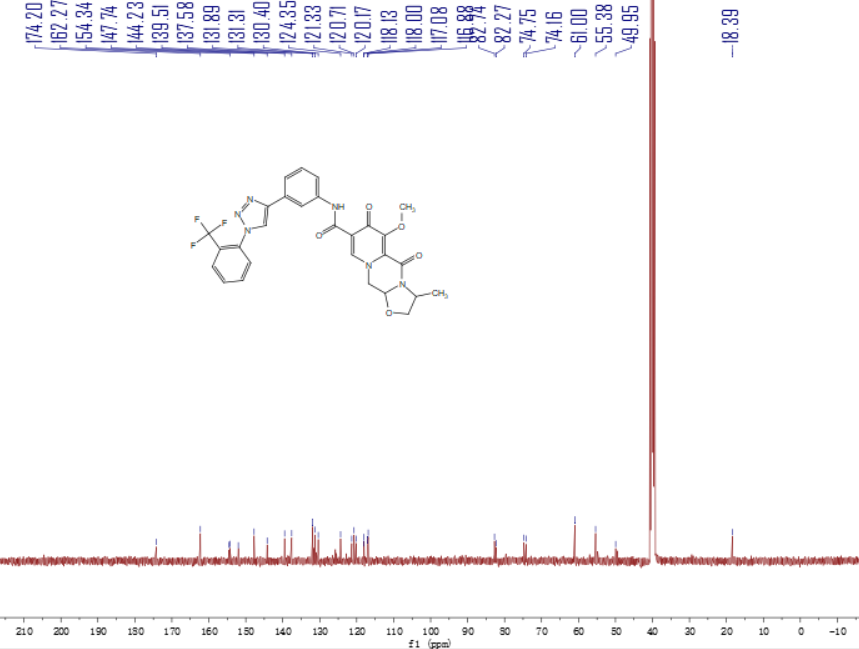


# Figure S2. ^1^H NMR and ^13^C NMR spectrums of compound 5b


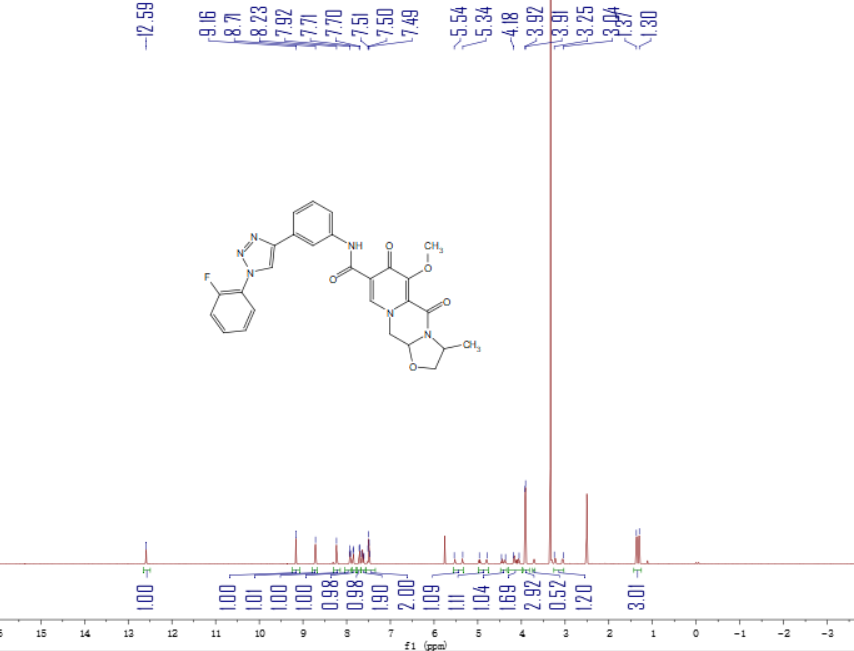


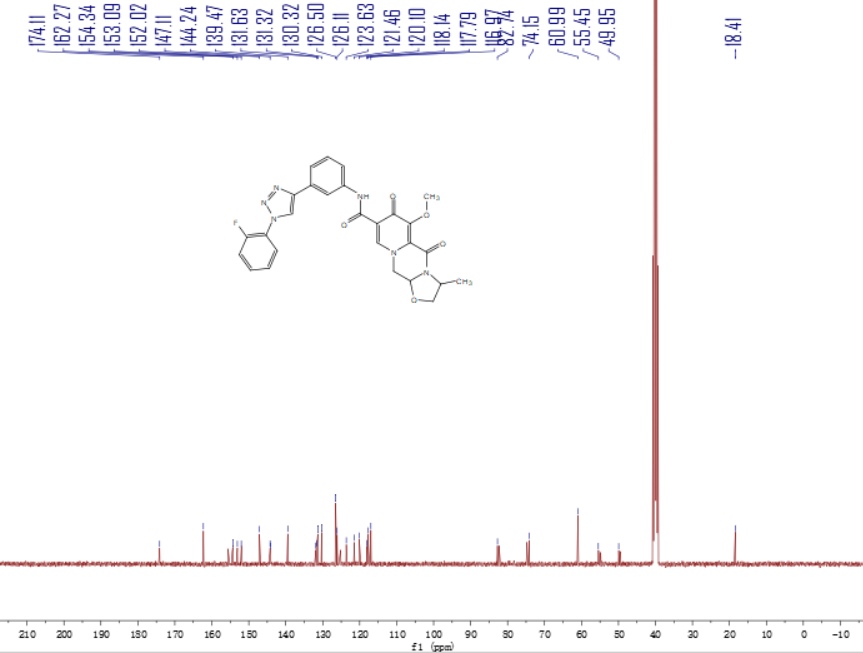


# Figure S3. ^1^H NMR and ^13^C NMR spectrums of compound 5c


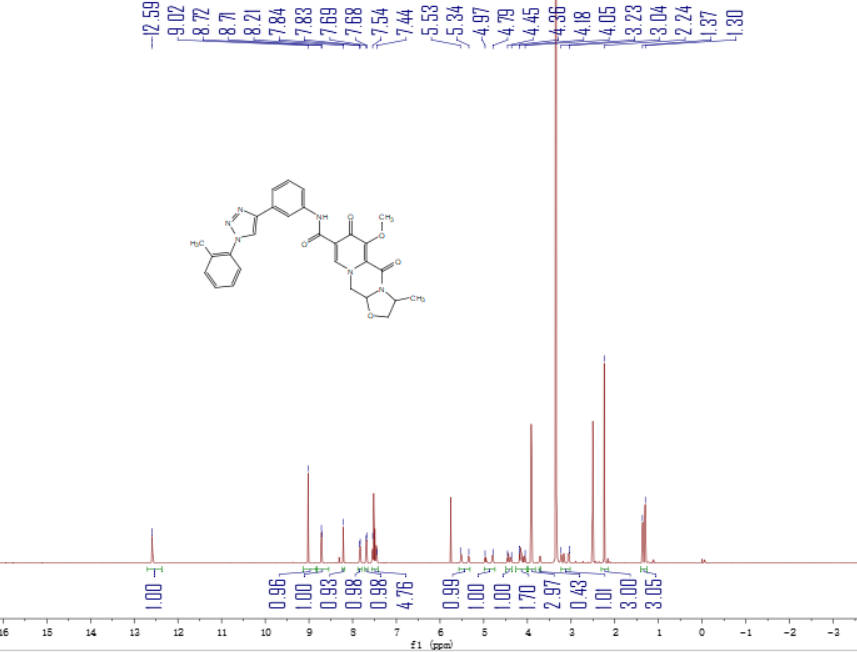


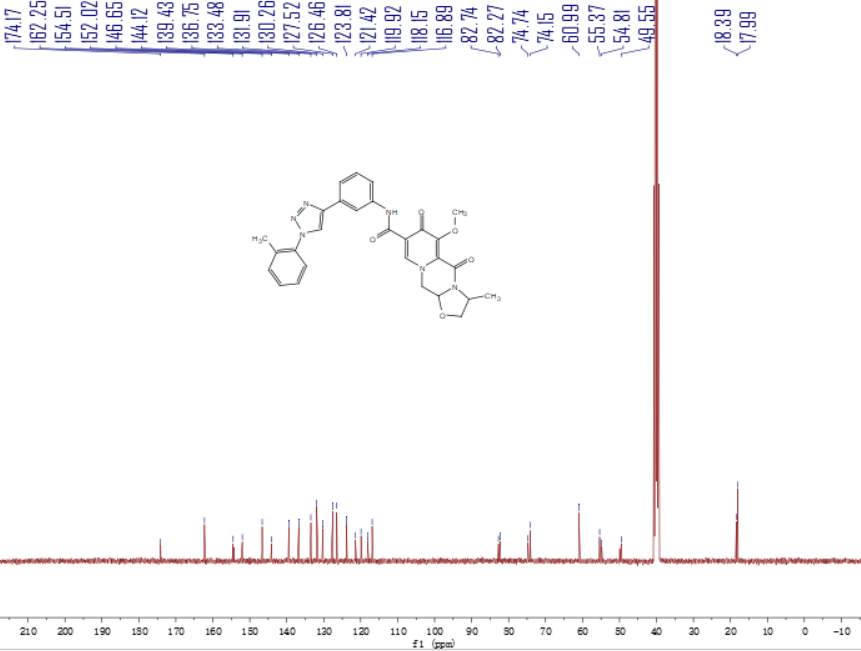


# Figure S4. ^1^H NMR and ^13^C NMR spectrums of compound 5d


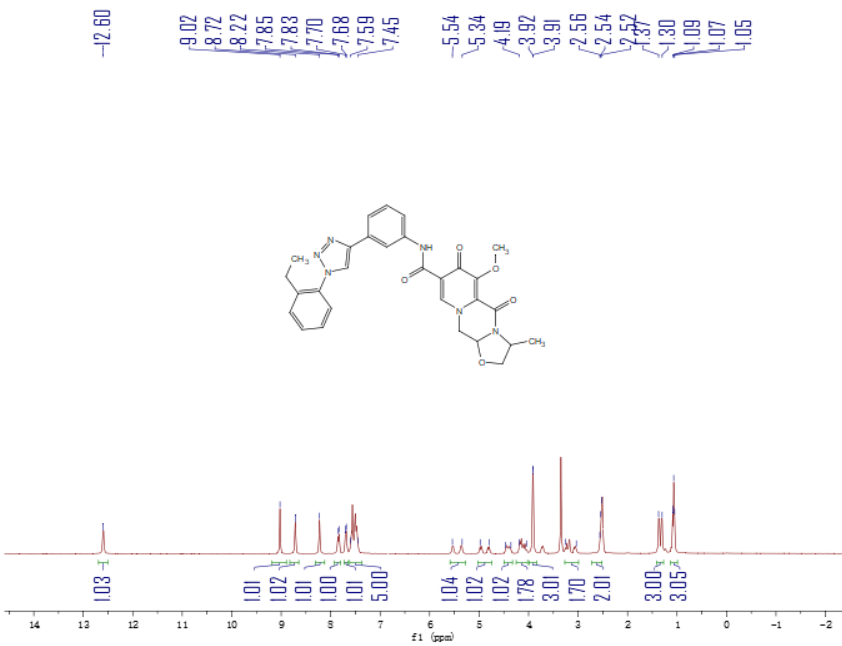


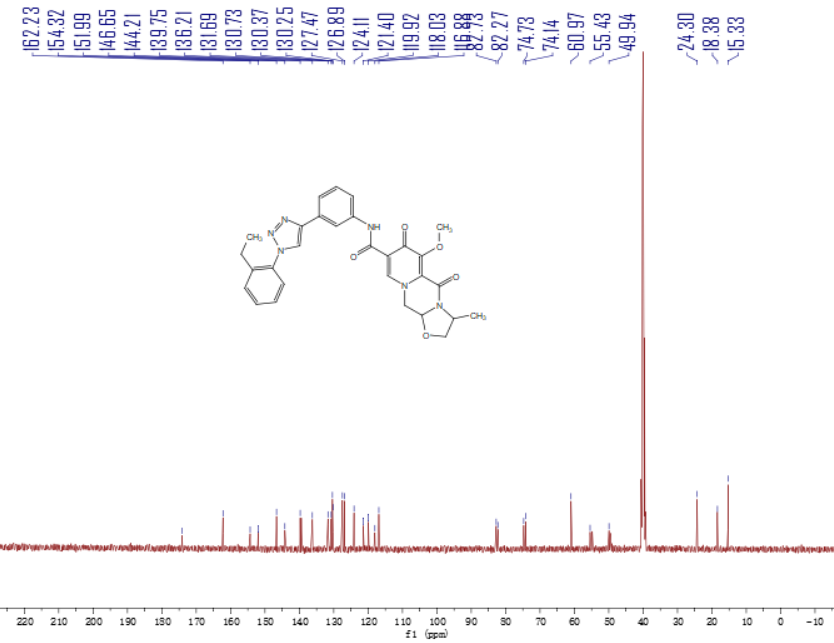


# Figure S5. ^1^H NMR and ^13^C NMR spectrums of compound 5e


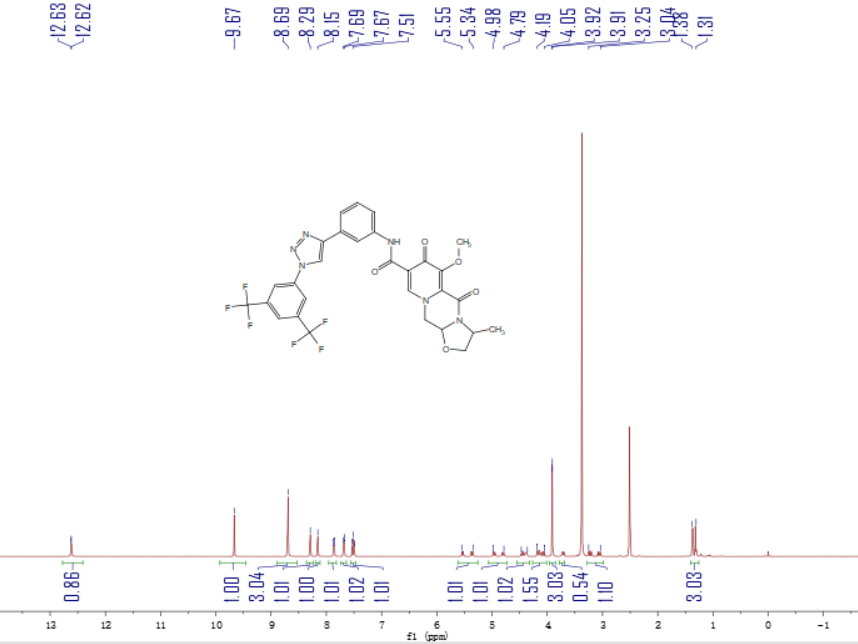


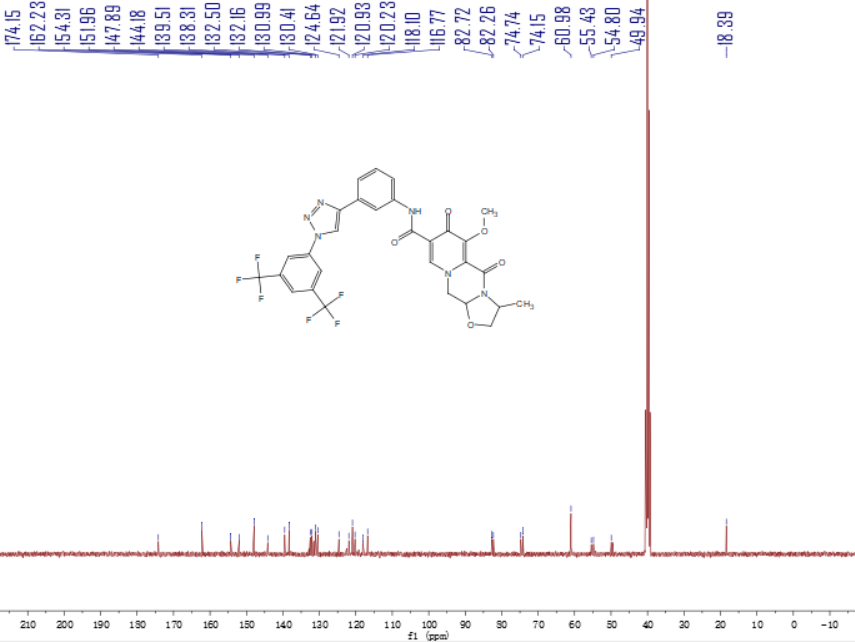


# Figure S6. ^1^H NMR and ^13^C NMR spectrums of compound 5f


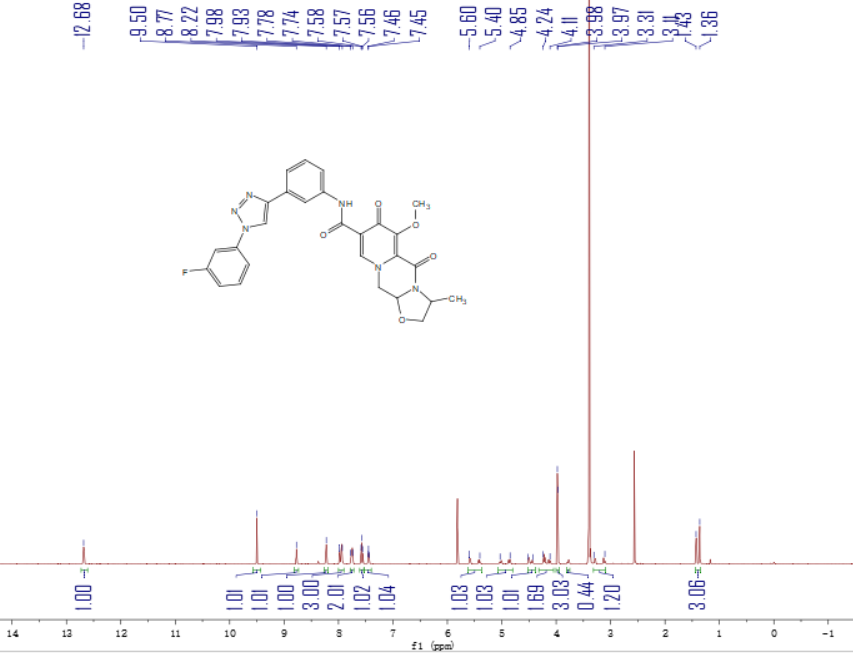


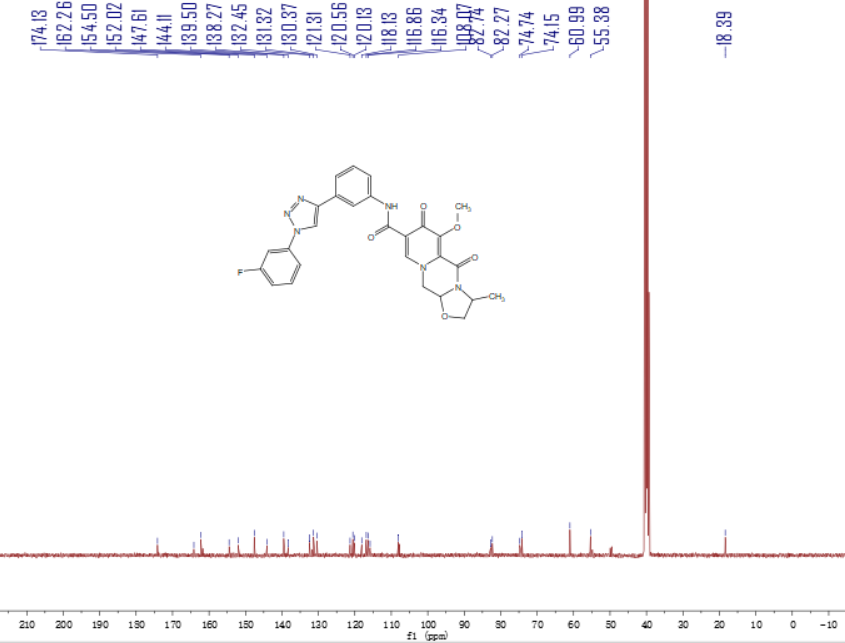


# Figure S7. ^1^H NMR and ^13^C NMR spectrums of compound 5g


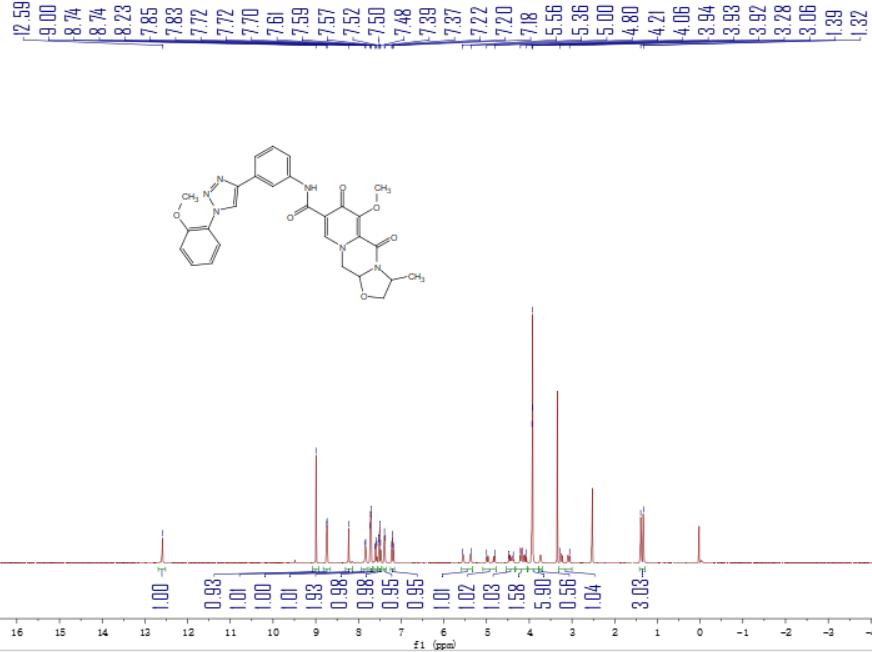


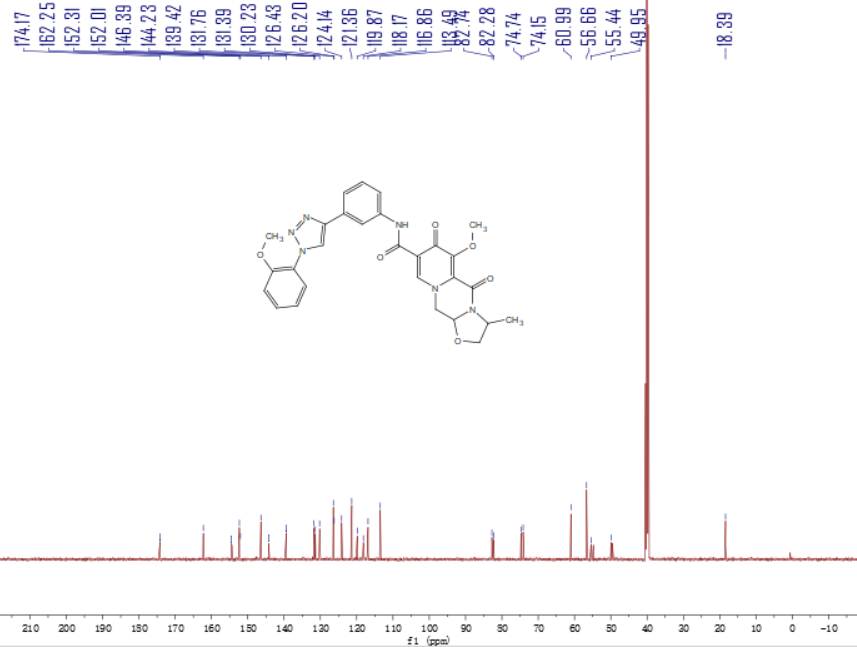


# Figure S8. ^1^H NMR and ^13^C NMR spectrums of compound 5h


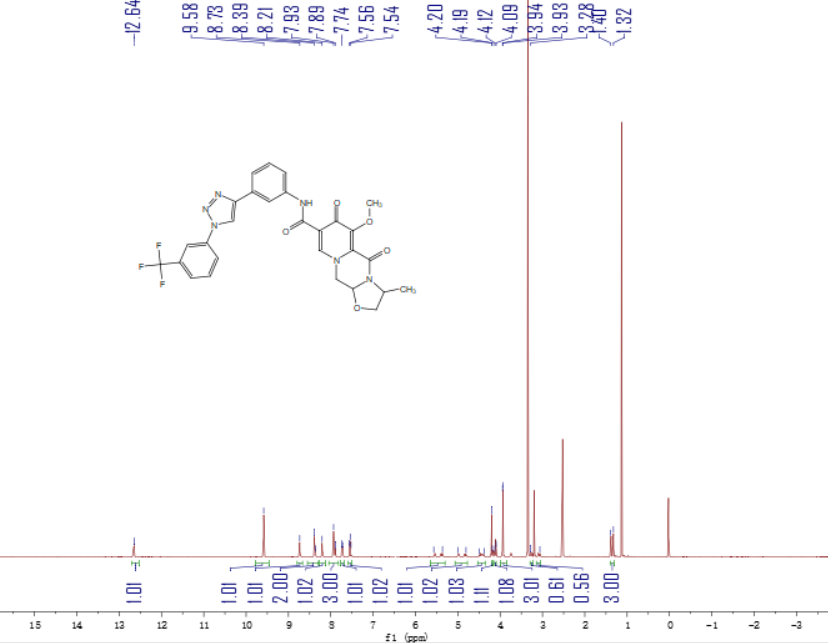


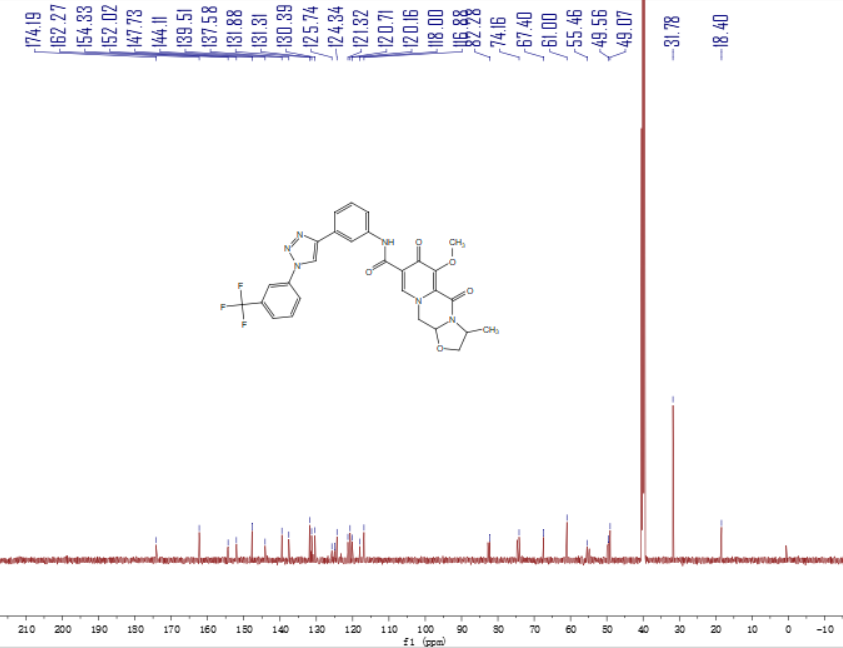


# Figure S9. ^1^H NMR and ^13^C NMR spectrums of compound 5i


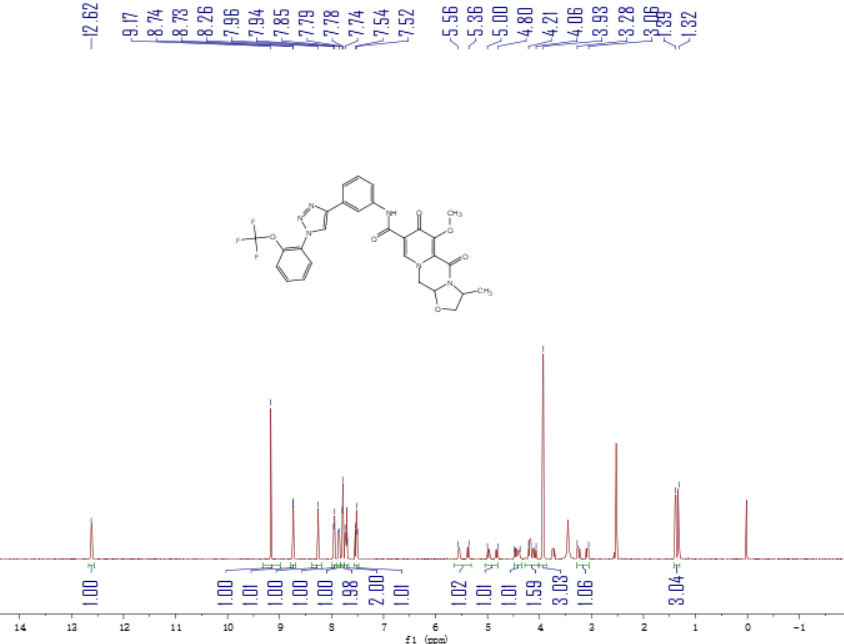


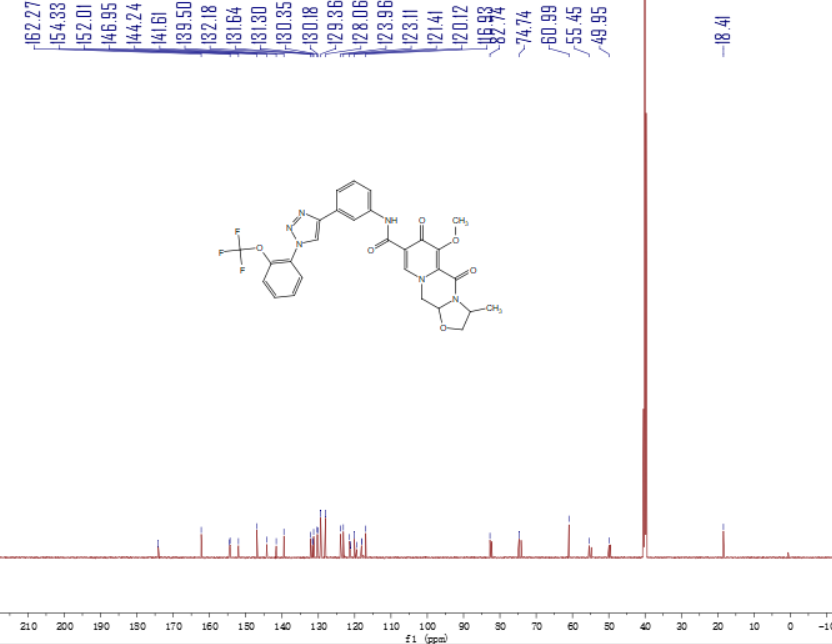


# Figure S10. ^1^H NMR and ^13^C NMR spectrums of compound 5j


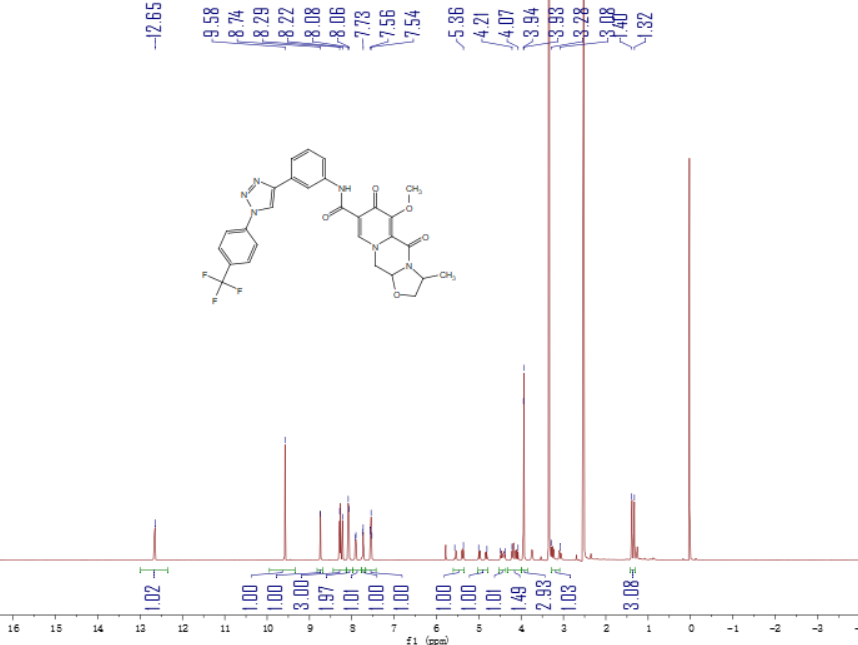


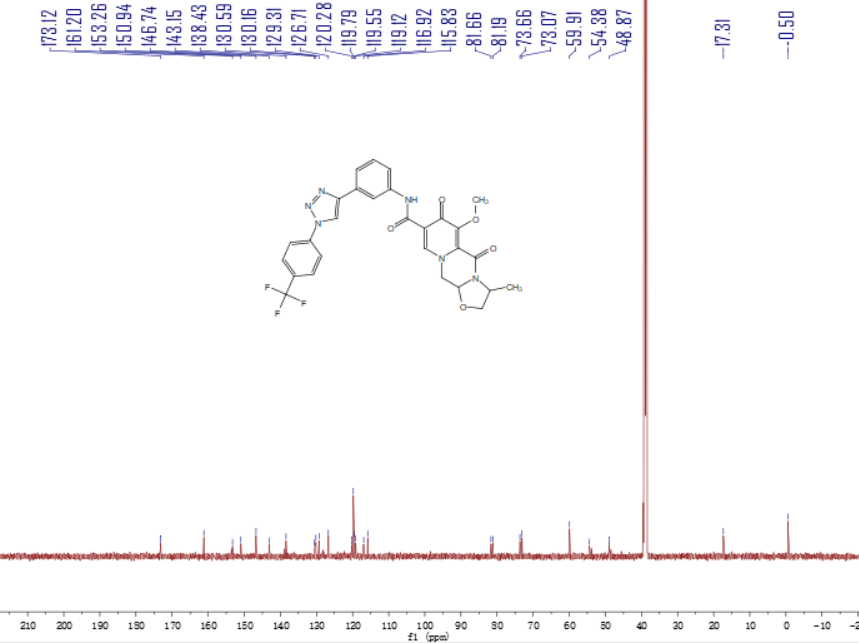


# Figure S11. ^1^H NMR and ^13^C NMR spectrums of compound 5k


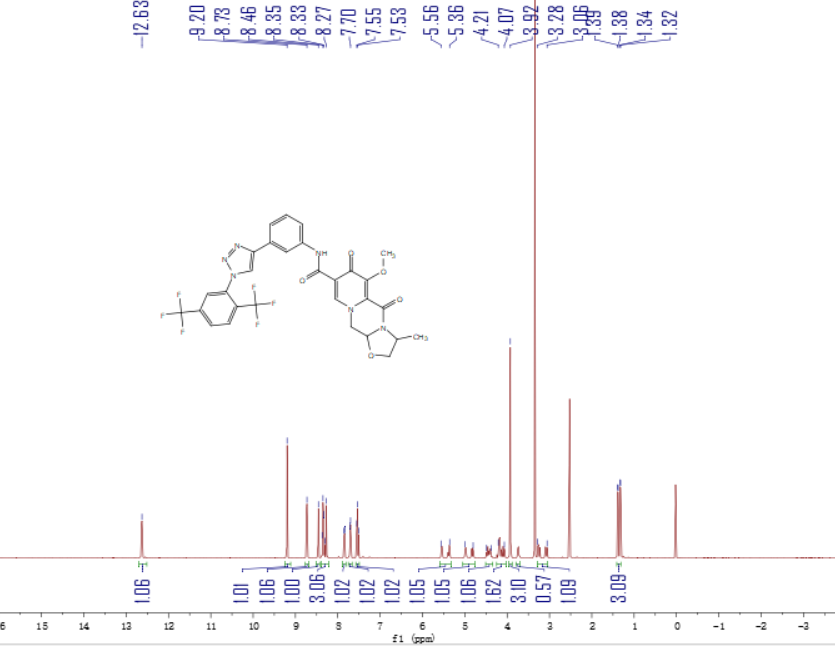


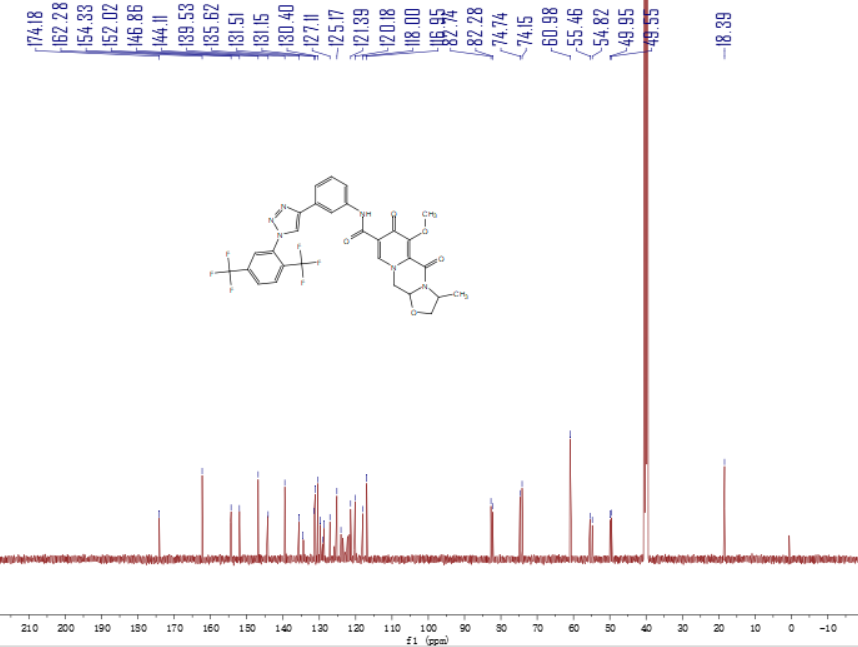


# Figure S12. ^1^H NMR and ^13^C NMR spectrums of compound 5l


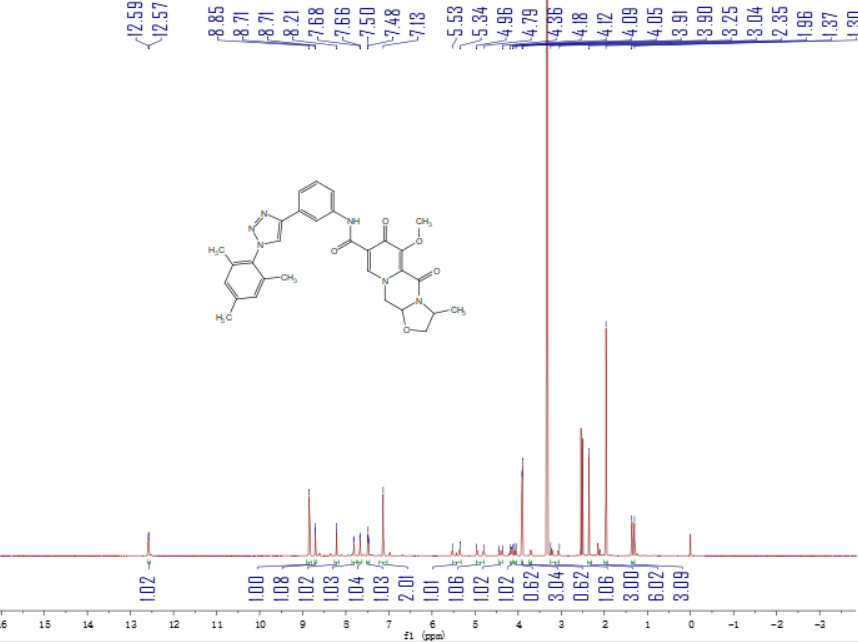


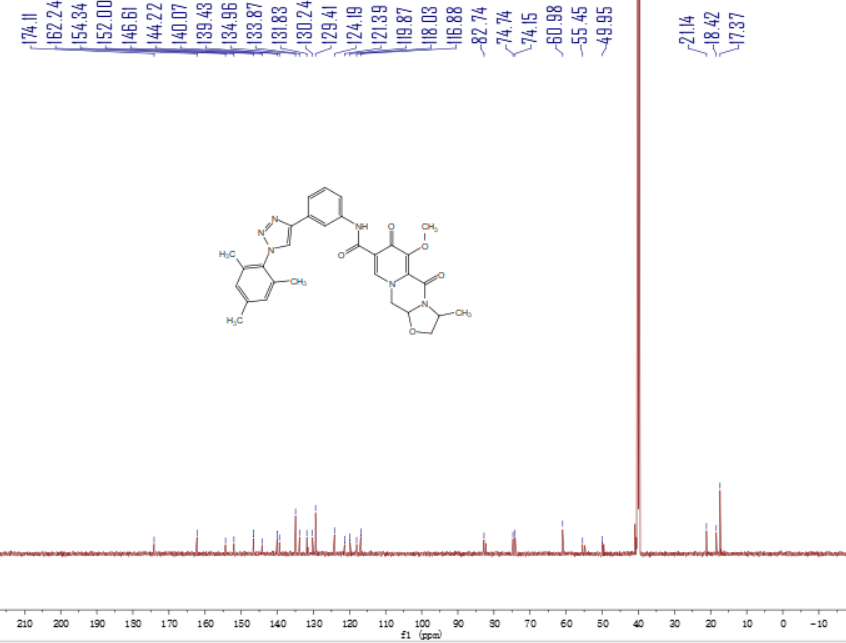


# Figure S13. ^1^H NMR and ^13^C NMR spectrums of compound 5m


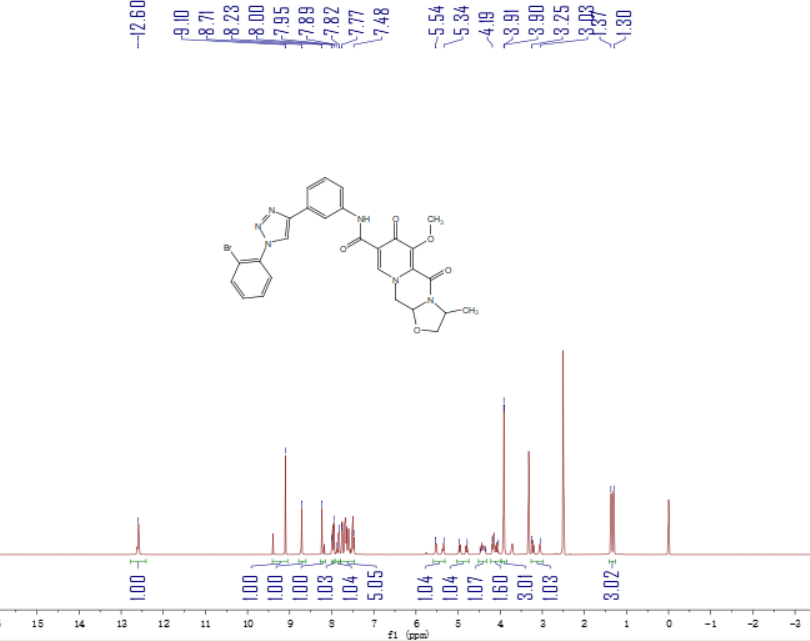


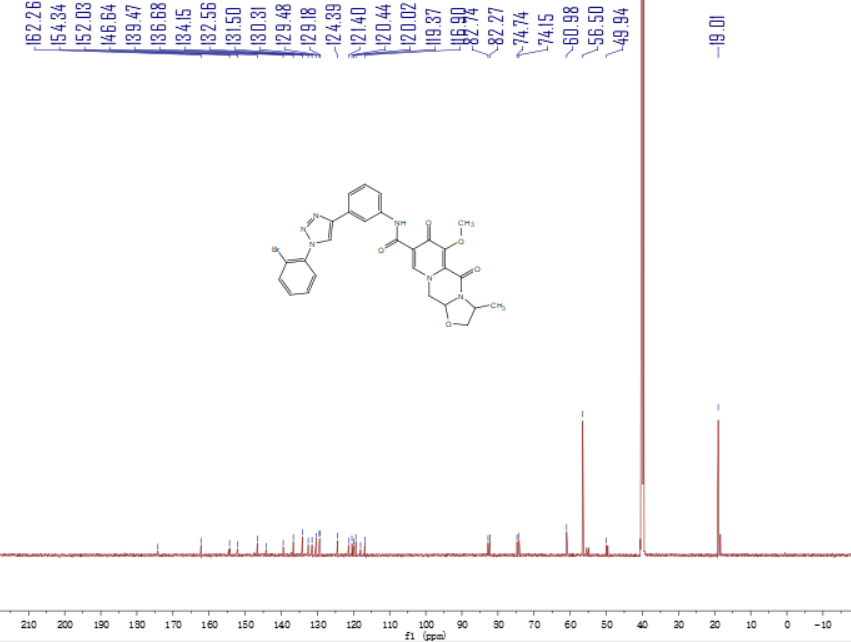


# Figure S14. ^1^H NMR and ^13^C NMR spectrums of compound 5n


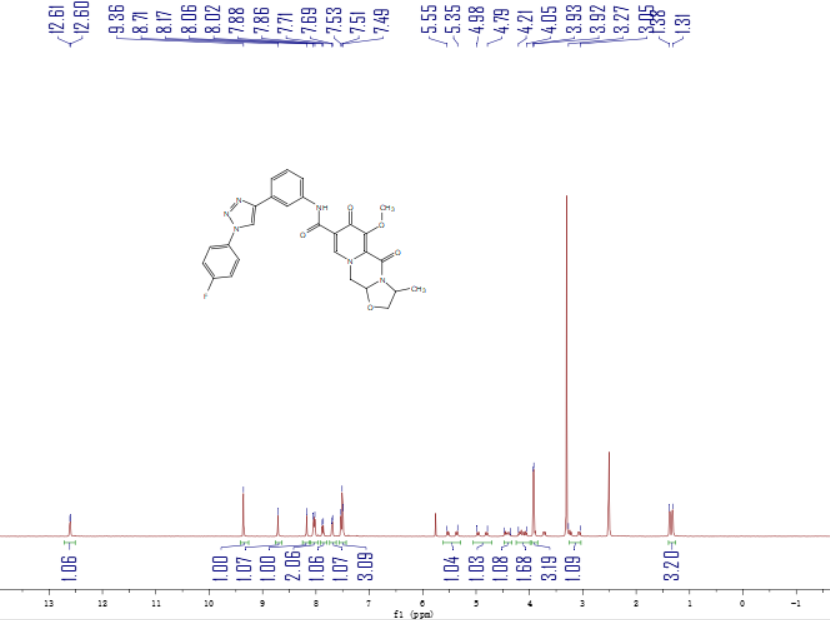


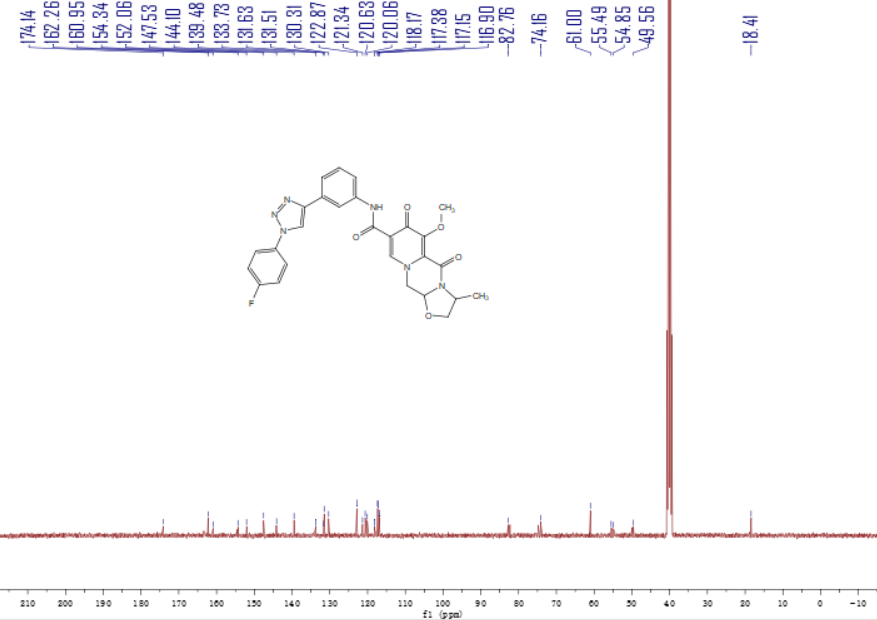


# Figure S15. ^1^H NMR and ^13^C NMR spectrums of compound 5o


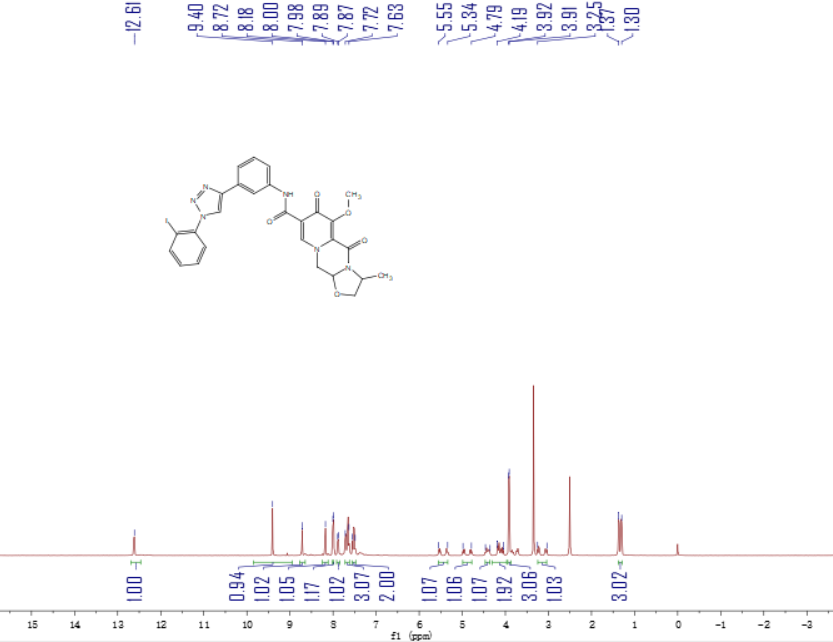


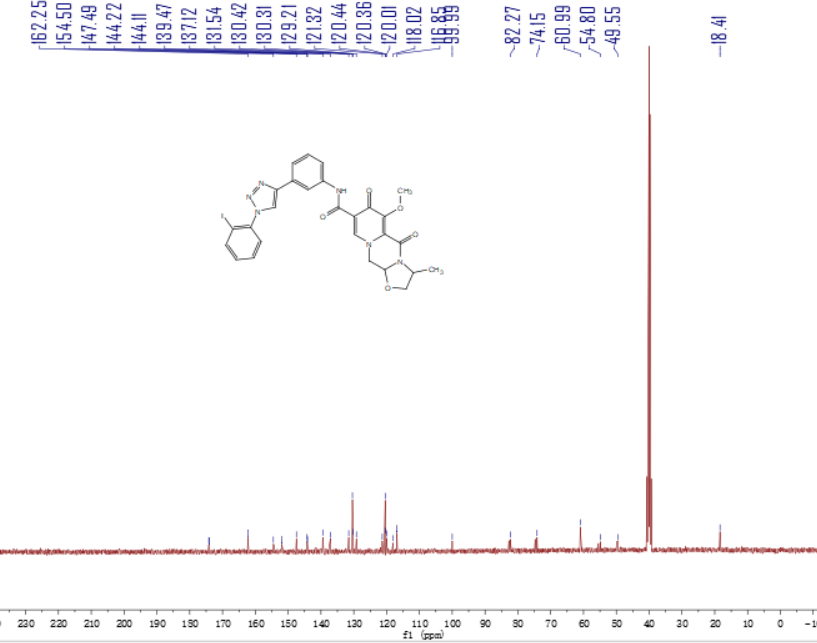


# Figure S16. ^1^H NMR and ^13^C NMR spectrums of compound 5p


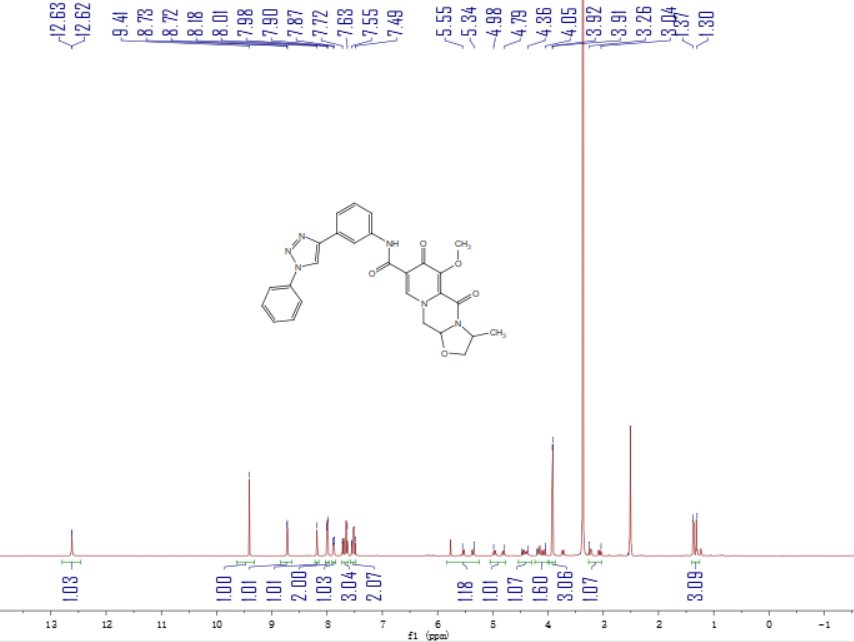


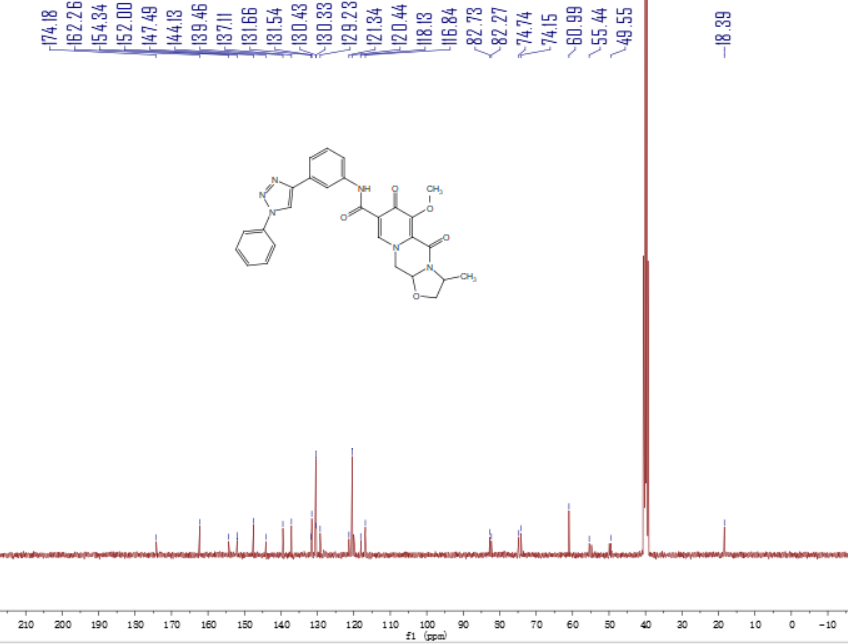


# Figure S17. ^1^H NMR and ^13^C NMR spectrums of compound 5q


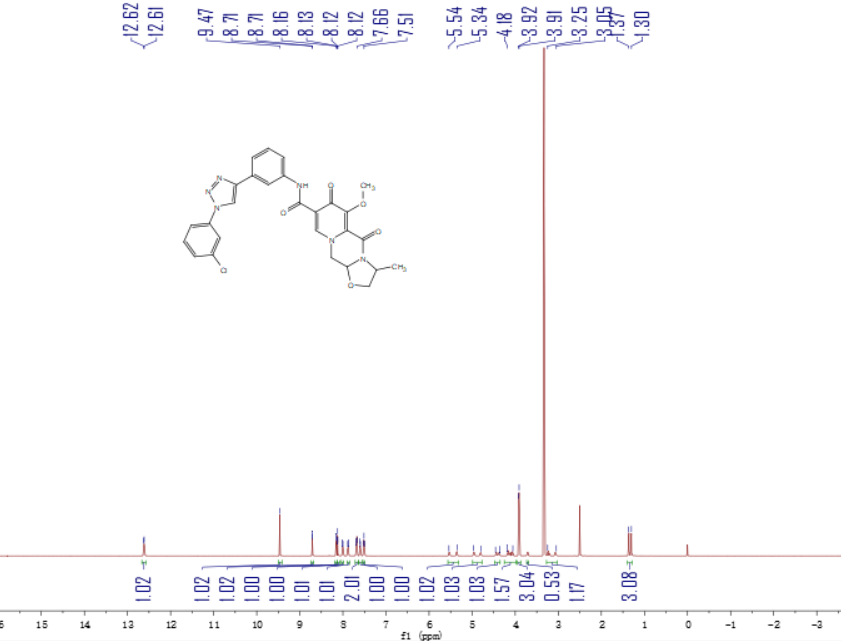


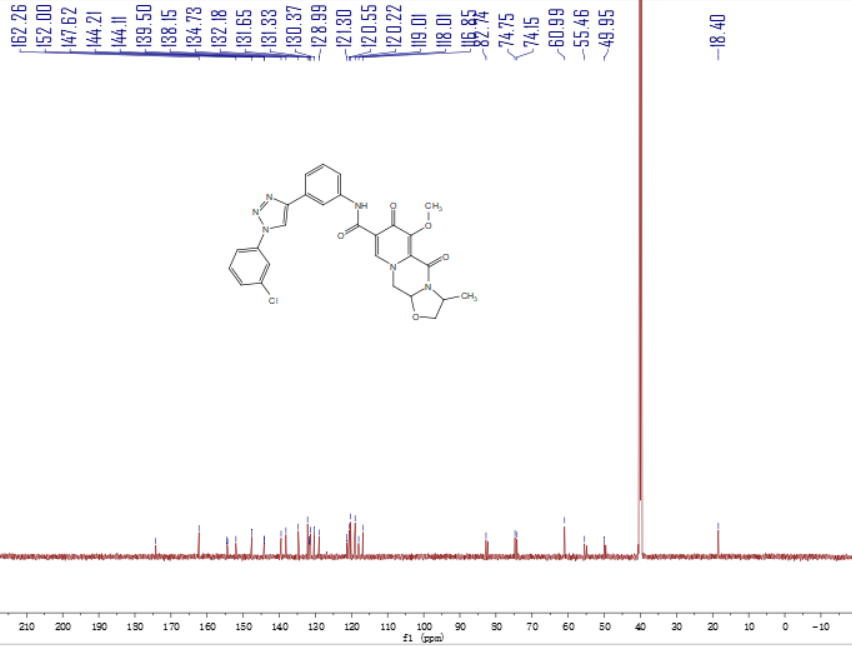


# Figure S18. ^1^H NMR and ^13^C NMR spectrums of compound 5r


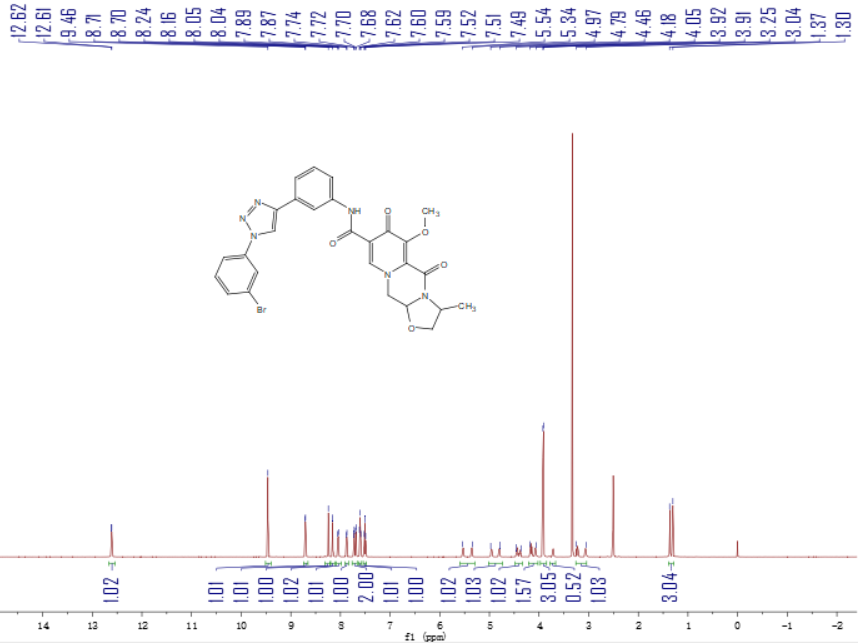


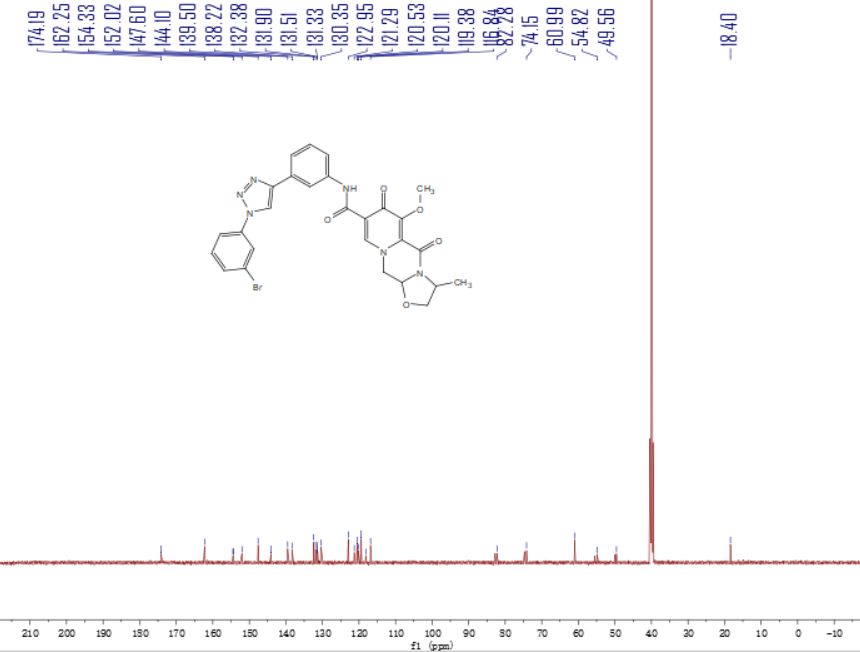


# Figure S19. ^1^H NMR and ^13^C NMR spectrums of compound 5s


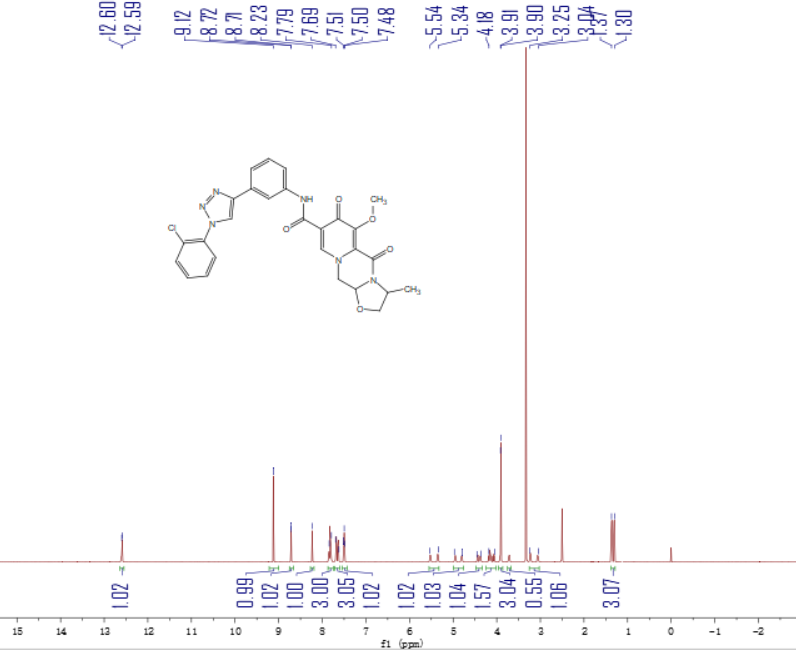


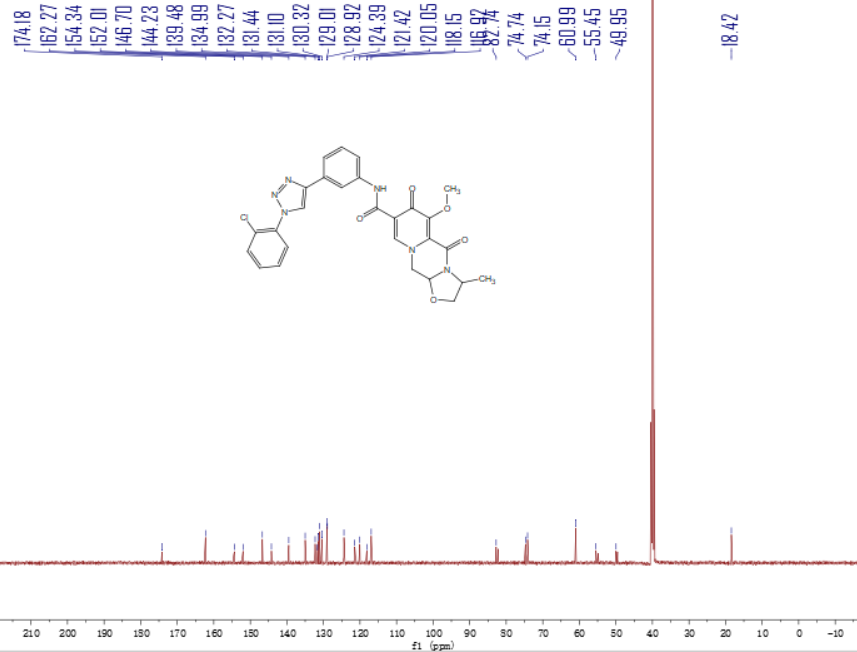


# Figure S20. ^1^H NMR and ^13^C NMR spectrums of compound 6a


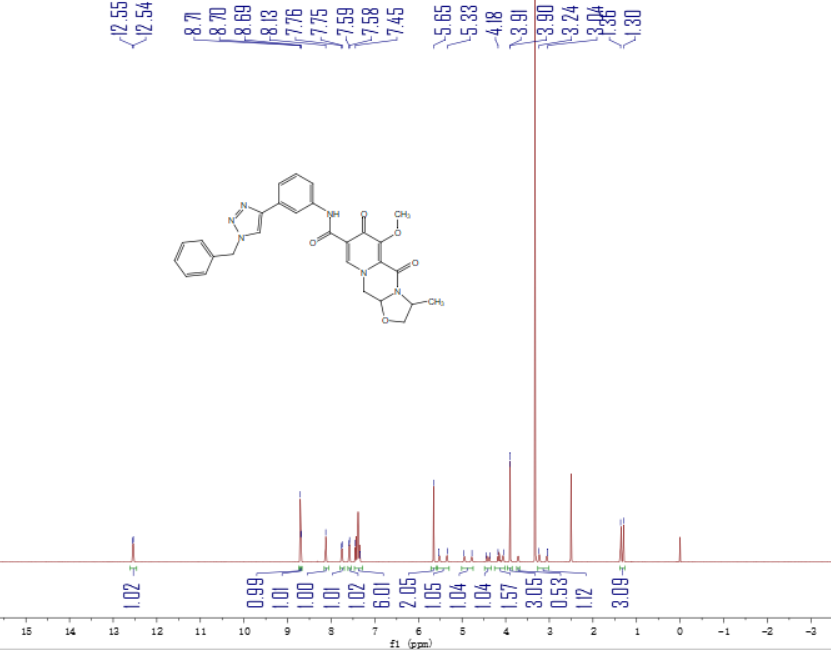


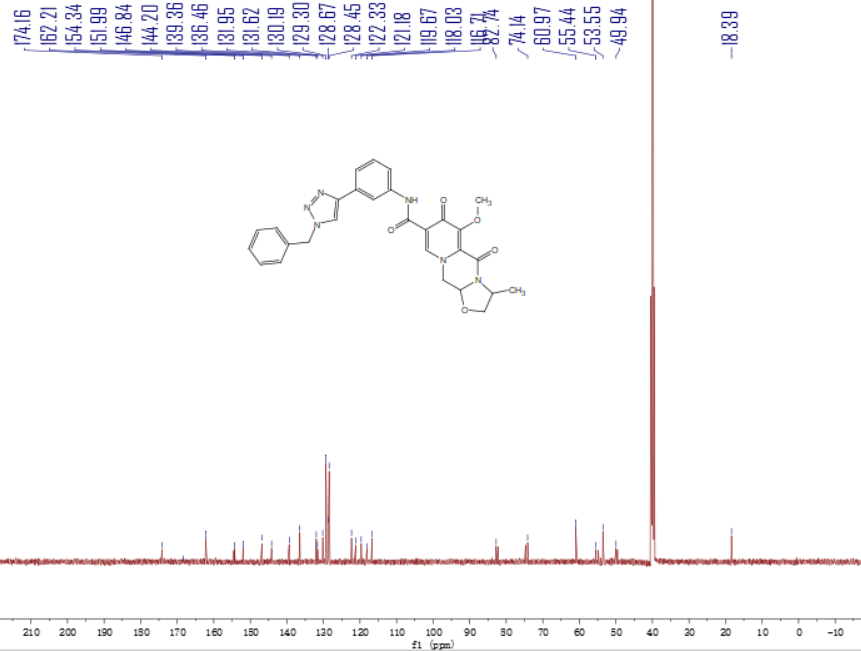


# Figure S21. ^1^H NMR and ^13^C NMR spectrums of compound 6b


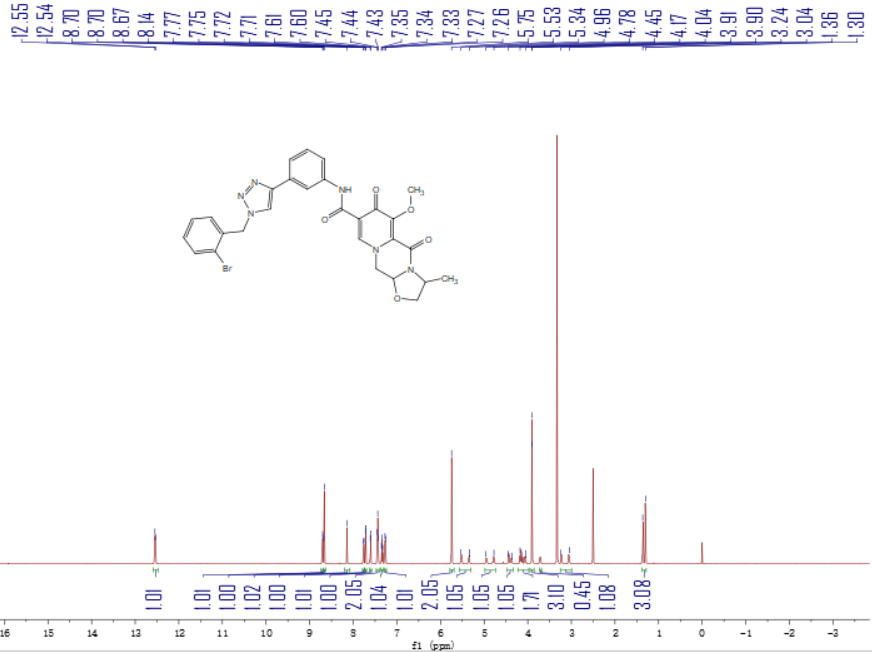


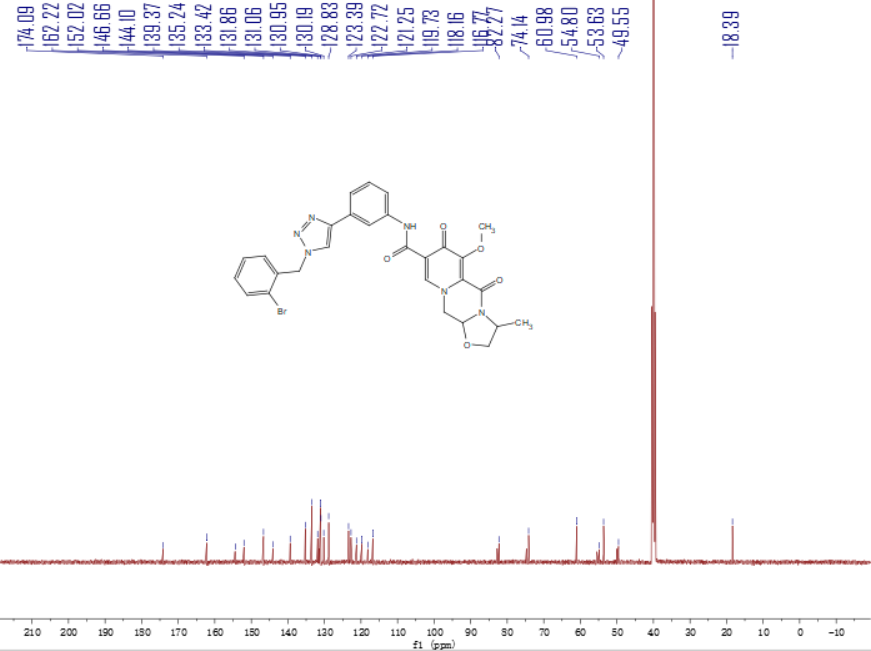


# Figure S22. ^1^H NMR and ^13^C NMR spectrums of compound 6c


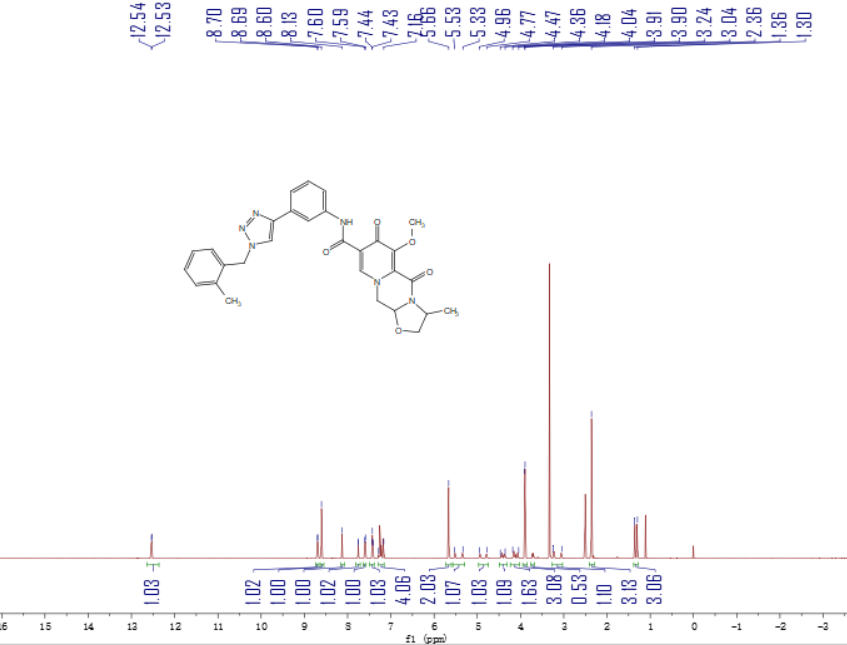


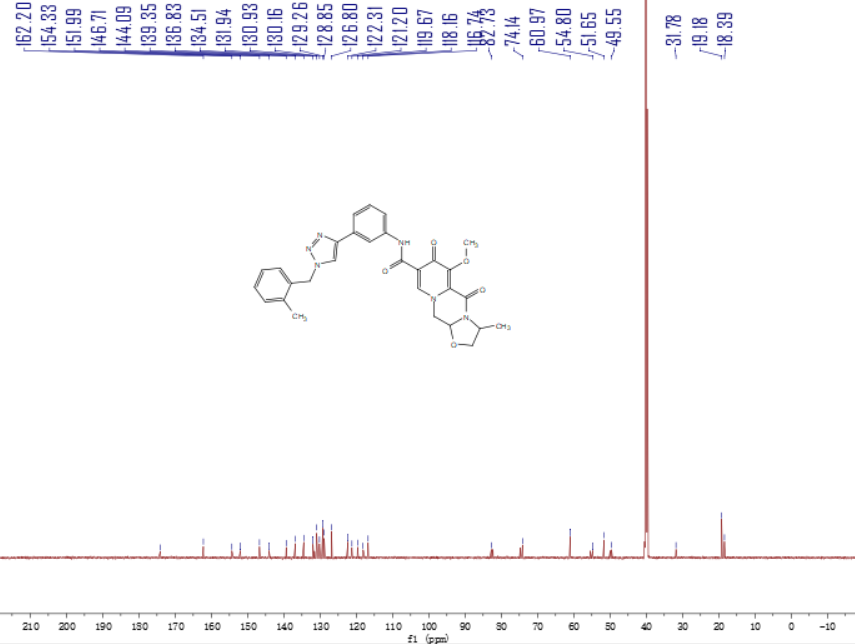


# Figure S23. ^1^H NMR and ^13^C NMR spectrums of compound 6d


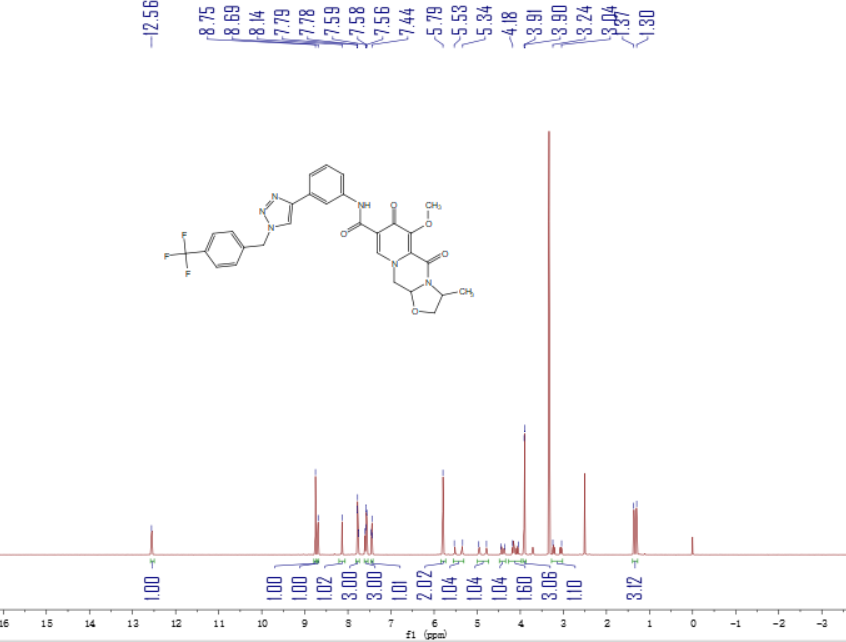


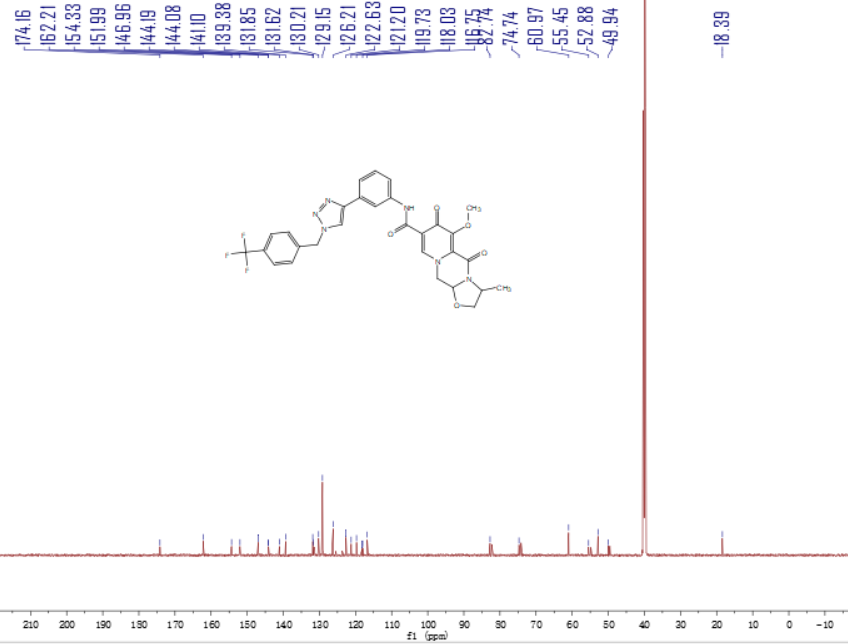


# Figure S24. ^1^H NMR and ^13^C NMR spectrums of compound 6e


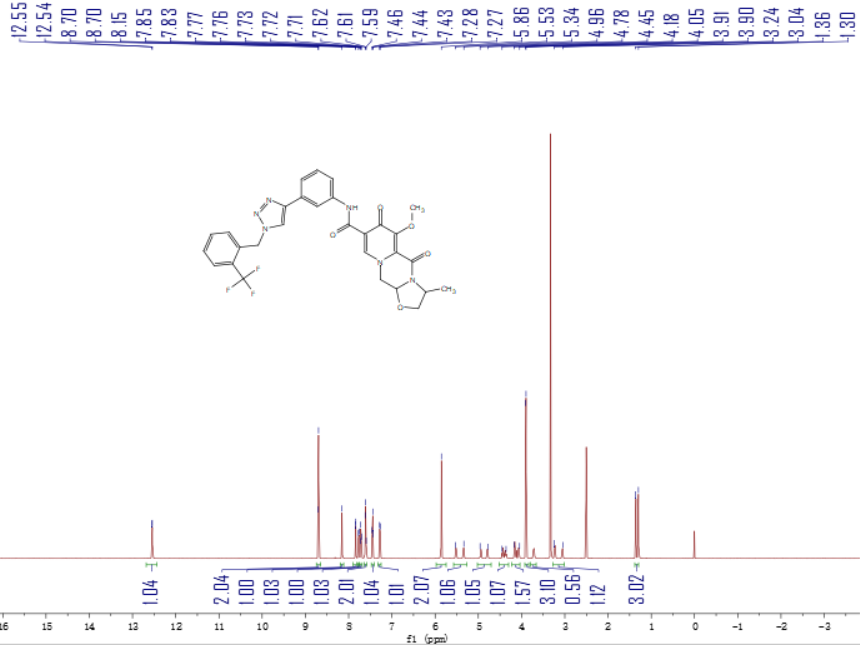


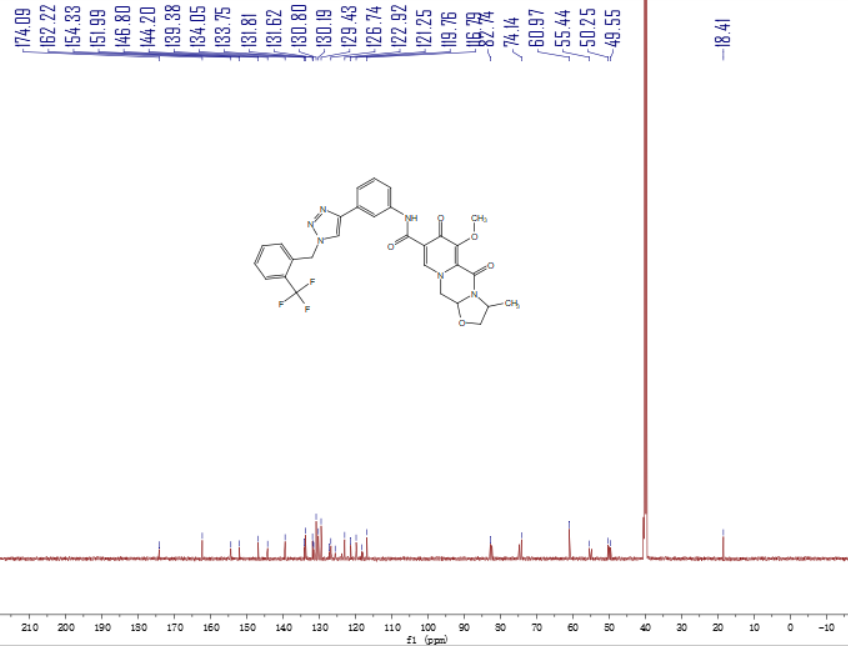


# Figure S25. ^1^H NMR and ^13^C NMR spectrums of compound 6f


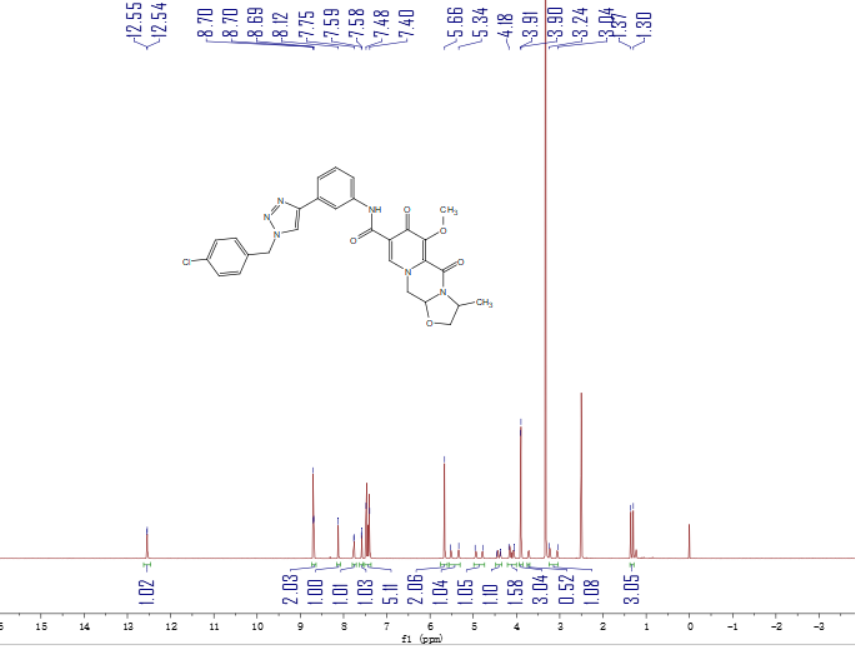


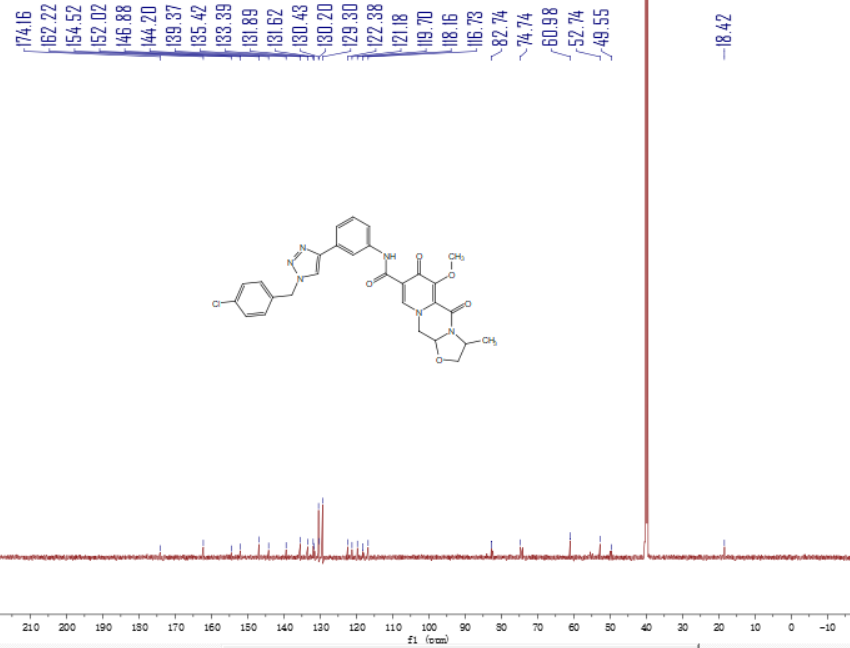


# Figure S26. ^1^H NMR and ^13^C NMR spectrums of compound 6g


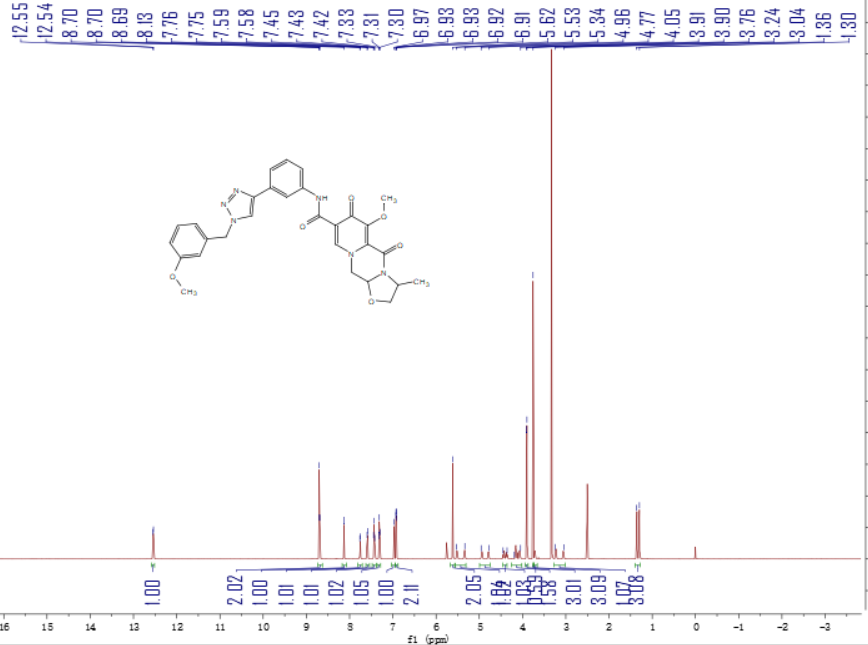


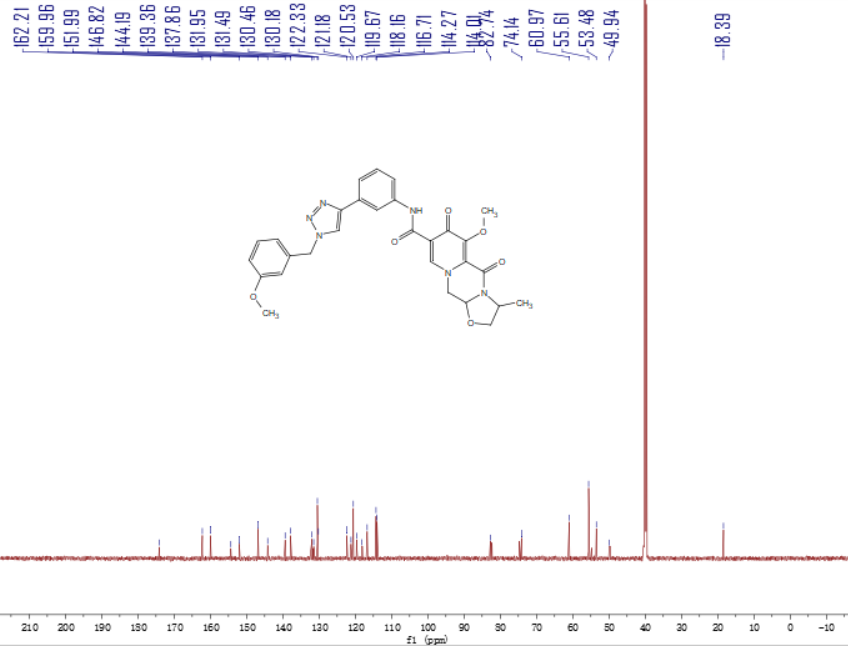


# Figure S27. ^1^H NMR and ^13^C NMR spectrums of compound 6h


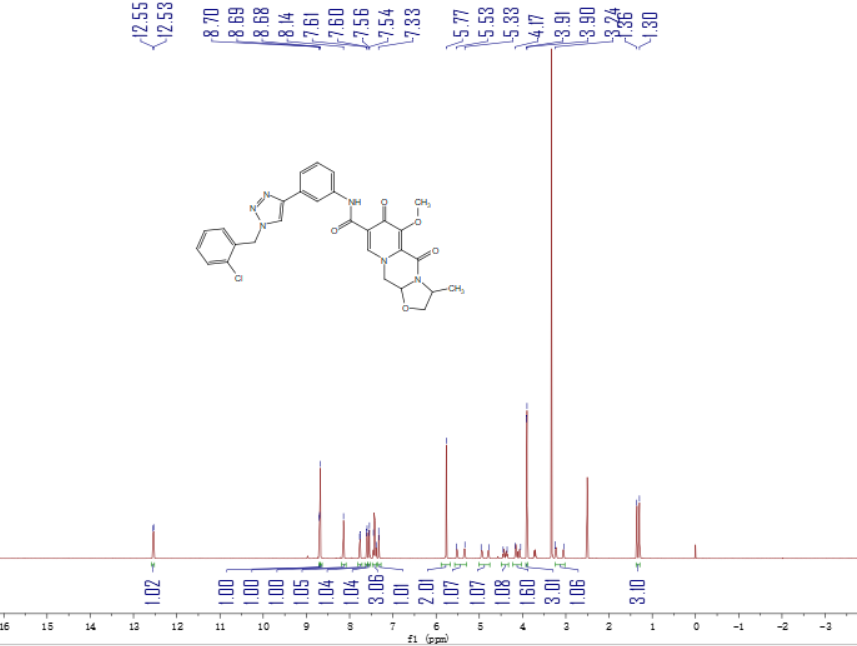


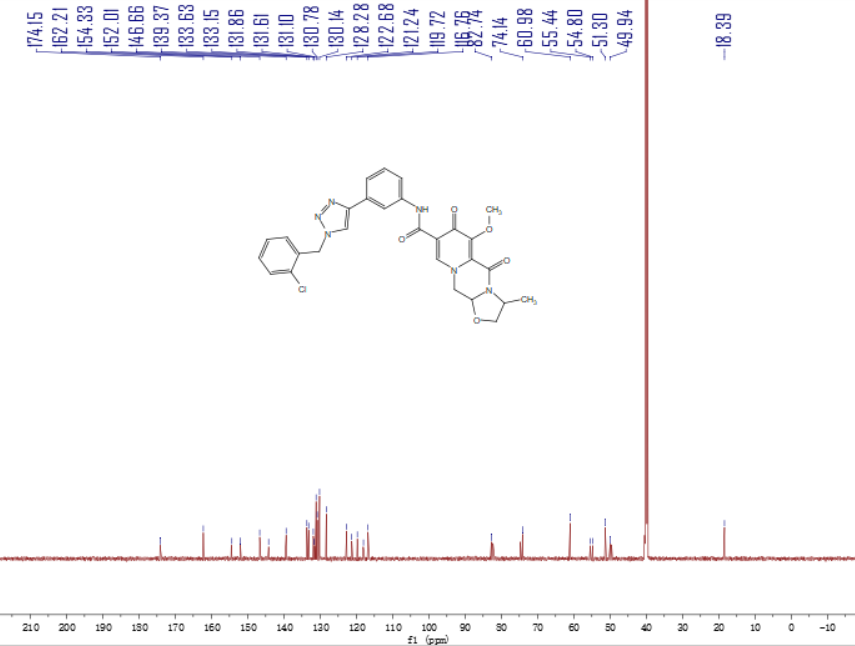


# Figure S28. ^1^H NMR and ^13^C NMR spectrums of compound 6i


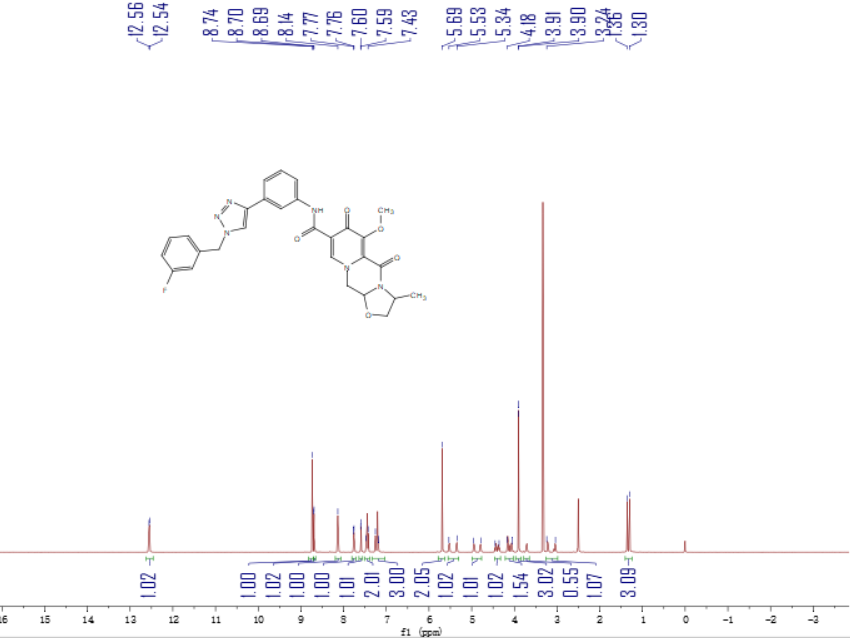


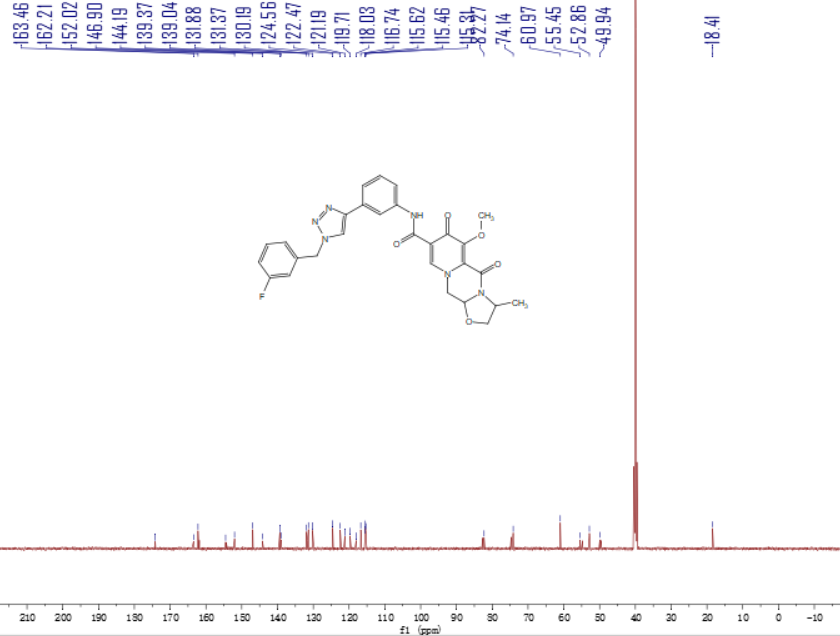

Supplement: Supplementary file 1 [file DataSheet1.docx]
